# Persistent Neurovascular Unit Dysfunction: Pathophysiological Substrate and Trigger for Late-Onset Neurodegeneration After Traumatic Brain Injury

**DOI:** 10.3389/fnins.2020.00581

**Published:** 2020-06-09

**Authors:** Yunxiang Zhou, Qiang Chen, Yali Wang, Haijian Wu, Weilin Xu, Yuanbo Pan, Shiqi Gao, Xiao Dong, John H. Zhang, Anwen Shao

**Affiliations:** ^1^Department of Surgical Oncology, The Second Affiliated Hospital, School of Medicine, Zhejiang University, Hangzhou, China; ^2^Department of Neurosurgery, The Second Affiliated Hospital, School of Medicine, Zhejiang University, Hangzhou, China; ^3^Department of Physiology and Pharmacology, Basic Sciences, School of Medicine, Loma Linda University, Loma Linda, CA, United States; ^4^Department of Anesthesiology, Neurosurgery and Neurology, School of Medicine, Loma Linda University, Loma Linda, CA, United States

**Keywords:** traumatic brain injury, neurodegenerative disease, neurovascular unit, hypothesis, neuroinflammation, proteinopathy, oxidative stress, blood–brain barrier

## Abstract

Traumatic brain injury (TBI) represents one of the major causes of death worldwide and leads to persisting neurological deficits in many of the survivors. One of the most significant long-term sequelae deriving from TBI is neurodegenerative disease, which is a group of incurable diseases that impose a heavy socio-economic burden. However, mechanisms underlying the increased susceptibility of TBI to neurodegenerative disease remain elusive. The neurovascular unit (NVU) is a functional unit composed of neurons, neuroglia, vascular cells, and the basal lamina matrix. The key role of NVU dysfunction in many central nervous system diseases has been revealed. Studies have proved the presence of prolonged structural and functional abnormalities of the NVU after TBI. Moreover, growing evidence suggests impaired NVU function is also implicated in neurodegenerative diseases. Therefore, we propose the Neurovascular Unit Dysfunction (NVUD) Hypothesis, in which the persistent NVU dysfunction is thought to underlie the development of post-TBI neurodegeneration. We deduce NVUD Hypothesis through relational inference and supporting evidence, and suggest continued NVU abnormalities following TBI serve as the pathophysiological substrate and trigger yielding chronic neuroinflammation, proteinopathies and oxidative stress, consequently leading to the progression of neurodegenerative diseases. The NVUD Hypothesis may provide potential treatment and prevention strategies for TBI and late-onset neurodegenerative diseases.

## Introduction

Traumatic brain injury (TBI) represents one of the major causes of mortality and disability worldwide, with a global burden of approximately US$ 400 billion annually (GBD 2016; Maas et al., 2017; de la Tremblaye et al., 2018; GBD 2016 Traumatic Brain Injury and Spinal Cord Injury Collaborators, 2019). Patients surviving the TBI usually suffer from various long-term neurological and neuropsychiatric sequelae, which include (but not limited to) neurodegenerative diseases (Cruz-Haces et al., 2017) and sleep disturbances (Castriotta et al., 2007; Barshikar and Bell, 2017). Actually, TBI-induced neurodegenerative disease was first introduced in the 19th century in professional boxers who repeatedly suffered head trauma (Martland, 1928). From then on, accumulating evidence has suggested that TBI is a significant risk factor for a variety of neurodegenerative diseases such as Alzheimer’s disease (AD) (Mortimer et al., 1991; Fleminger et al., 2003; Hayes et al., 2017; LoBue et al., 2019), Parkinson’s disease (PD) (Goldman et al., 2006; Jafari et al., 2013), and amyotrophic lateral sclerosis (ALS) (Chen et al., 2007; Franz et al., 2019). Notably, sleep deprivation itself is an important predisposing factor for the development of neurodegenerative diseases (Boespflug and Iliff, 2018; Shokri-Kojori et al., 2018; Cordone et al., 2019).

Neurodegenerative diseases are declared a group of chronic diseases burdening the global aging society (de Lau and Breteler, 2006; Li et al., 2013). Alzheimer’s disease is considered the most common neurodegenerative disorders, followed by PD. In 2015, the former incurred worldwide losses totaling US$ 818 billion, an increase of 35% over the previous five years (Fotuhi et al., 2009; Masters et al., 2015; Ascherio and Schwarzschild, 2016; Wimo et al., 2017). Although symptomatic and etiological treatments targeting patients with AD or PD may help alleviate some of the physical or mental symptoms of these incurable, age-related diseases, no treatment strategies have been proved sufficiently effective thus far (Ascherio and Schwarzschild, 2016; Kumar et al., 2016; Mendiola-Precoma et al., 2016; Orimo, 2017). Another neurodegenerative disease, ALS, although relatively less frequent, has a high fatality rate and a median survival of only 14 months from the time of diagnosis (Luna et al., 2019). Therefore, considering the huge medical and social burden of neurodegenerative diseases and the increased incidence in patients with prior TBI, exploring the underlying mechanisms of causality between TBI and post-TBI neurodegenerative diseases is of great significance.

**FIGURE 1:**
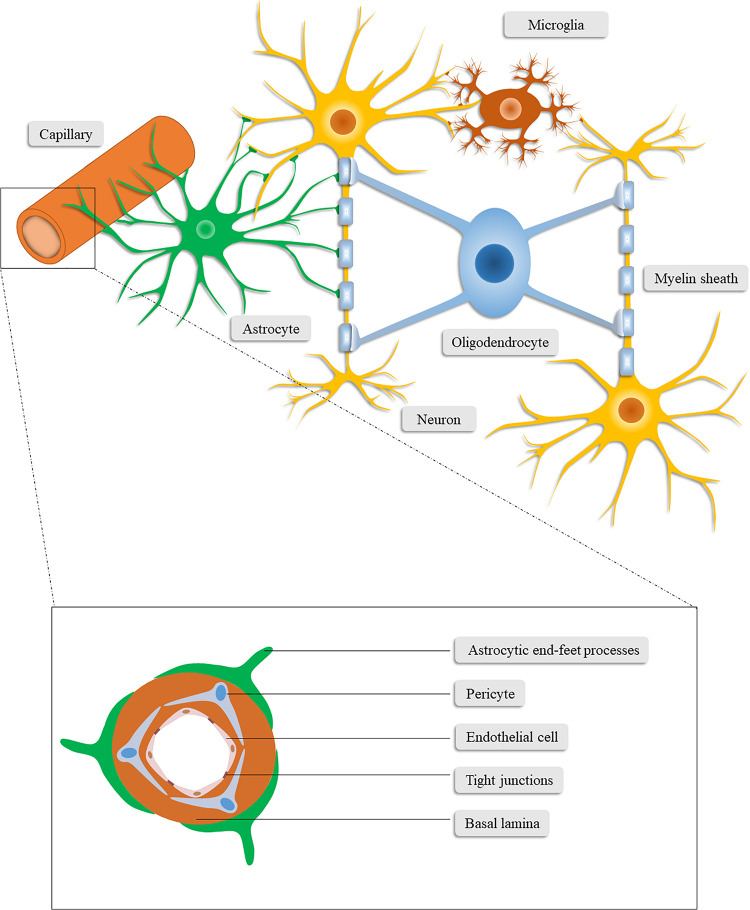
Schematic illustration of the neurovascular unit. The neurovascular unit consists of neurons, neuroglia (astrocytes, microglia, and oligodendrocytes), vascular cells (pericytes, endothelial cells, and vascular smooth muscle cells) and the basal lamina matrix of brain vasculature.

The neurovascular unit (NVU; Figure 1) was originally introduced as a conceptual framework for cerebrovascular diseases, especially ischemic stroke, and has recently been identified as a key player in many other central nervous system (CNS) diseases, including TBI, whose primary and secondary pathologic processes can lead to persistent structural or metabolic abnormalities of the NVU (Zhang et al., 2005; Lok et al., 2015; Perez et al., 2017). Recently, more and more lines of evidence have proved that NVU dysfunction is also related to neurodegenerative diseases (Cai et al., 2017b). Thus, NVU dysfunction participates in both TBI and neurodegenerative diseases, and it may be a candidate mechanism underlying the TBI-induced neurodegenerative diseases. To understand the underlying mechanisms further, we propose the Neurovascular Unit Dysfunction (NVUD) Hypothesis, in which persistent NVU dysfunction accounts for the pathophysiological substrate and trigger condition for post-TBI neurodegeneration. In the present study, we elaborate on the functions of the components of the NVU and their significant roles in the pathophysiology of TBI. We also present scientific evidence supporting the involvement of NVU dysfunction in neurodegenerative disorders and attempt to summarize precise mechanisms linking acute TBI to chronic effects on neurodegeneration. Consequently, we suggest the reasonability of the NVUD Hypothesis and illuminate its value and limitations.

## The Components of the NVU Play Critical Roles in the Homeostasis of the CNS

The paradigm of the NVU nowadays encompasses neurons, neuroglia (astrocytes, microglia, and oligodendrocytes), vascular cells (pericytes, endothelial cells, and vascular smooth muscle cells), and the basal lamina matrix of brain vasculature (Lo and Rosenberg, 2009; Cai et al., 2017a). Among these components, neurons may constitute the most pivotal cells for neurological function and have been studied by a great deal of previous work (Cai et al., 2017b). In this section, we mainly focus on the components other than neurons, which also play critical roles in the homeostasis of the CNS and closely interact with neurons.

### Glial Components in the NVU

Brain-resident glial cells, which are comprised of astrocytes (astroglia), oligodendrocytes and microglia, exert various functions to maintain CNS homeostasis (Sofroniew and Vinters, 2010; Yang and Wang, 2015). Specifically, astrocytes, the most abundant brain-resident glial cells (Yang and Wang, 2015), stretch out their endfeet to the microvessels to regulate cerebral blood flow and form a functional barrier named glia limitans (Abbott et al., 2006). Through the glia limitans, astrocytes separate neurons from the blood vessels, meninges and perivascular spaces (Sofroniew, 2015). In addition to structural support for neurons, astrocytes also functionally favor neurons in a number of ways (Zhou et al., 2020), including regulating the neurotransmitter glutamate (Rothstein et al., 1996; Anderson and Swanson, 2000; Zou et al., 2010), releasing neurotrophic factors and gliotransmitters (Ye et al., 2003; Kardos et al., 2017; Perez et al., 2017), synthesizing glutamine, cholesterol, glutathione, and thrombospondin (Dringen et al., 2000; Slemmer et al., 2008; Colangelo et al., 2012), converting glucose into lactate (Magistretti and Pellerin, 1999; Danbolt, 2001; Magistretti, 2006), and controlling water homeostasis and neuronal activation (Lang et al., 1998; Walz, 2000; Kofuji and Newman, 2004; Jayakumar and Norenberg, 2010). Furthermore, the concentration of extracellular ions (Colangelo et al., 2012) and the glymphatic system (Jessen et al., 2015) are regulated by astrocytes.

Microglia are immunocompetent cells in the CNS that are easily activated and have the function of recognizing and phagocytizing pathogens, debris and dead cells. They form the first line of defense system of the CNS and conduct phagocytosis-mediated cleanup during senescence or pathological processes (Hu et al., 2014). Conversely, microglia are also involved in the secretion of neurotoxins, such as nitric oxide and pro-inflammatory cytokines, which further deteriorate the cerebral microenvironment in pathological conditions (Hailer, 2008).

Oligodendrocytes, another type of brain-resident glial cell, produce lipid-enriched myelin that wraps axons and accelerates nerve impulses (Plemel et al., 2014). Taken together, glial cells can affect the outcome of patients with CNS disorders given the multifunctional roles of glial cells in the CNS.

### Microvascular Components in the NVU

Vascular cells involved in the NVU include pericytes, endothelial cells, and vascular smooth muscle cells. As vascular wall cells embedded in the microvascular basement membrane, pericytes are centrally positioned between endothelial cells, astrocytes and neurons (Sweeney et al., 2016). Pericytes are involved in signal transduction and communication with neighboring cells, through which they regulate blood–brain barrier (BBB) permeability, angiogenesis, neurotoxic metabolism and clearance, neuroinflammation, capillary hemodynamic response, and stem cell activity, demonstrating their vital role in the CNS during health and disease (Sweeney et al., 2016).

Similar to pericytes, endothelial cells are physiologically versatile cells that are involved in the maintenance of vascular homeostasis, regulation of cerebral vasoconstriction and vasodilatation, angiogenesis, and secretion of anti-coagulation factors. Expressing multiple substrate-specific transport systems, endothelial cells also play a vital role in controlling the exchange of CNS with peripheral substances and regulating the transport of ions and nutrients (Cai et al., 2017b). Endothelial cells are highly connected through tight junctions (TJs) which restricts the paracellular diffusion of substances from blood to the CNS (Tso and Macdonald, 2014). These continuously interconnected endothelial cells, together with pericytes, the basal lamina matrix and the astrocytic end-foot processes, constitute the BBB (Najjar et al., 2017). The integrity of the BBB is critical to the homeostasis and immunoprotection of the CNS (Najjar et al., 2017). Of the microvascular components in the NVU, endothelial cells are the main component of the BBB (Cai et al., 2017b) while pericytes primarily maintain the integrity of the BBB (Armulik et al., 2010; Bell et al., 2010; Daneman et al., 2010).

### The Extracellular Matrix

The extracellular matrix (ECM) of the basal lamina functions as an anchor for the endothelium through cell-matrix interactions between laminin (or other matrix proteins) and the endothelial integrin receptors. Through these interactions, numerous intracellular signaling pathways can be stimulated (Hynes, 1992; Tilling et al., 2002). The TJs are thought to constitute the primary impediment of paracellular diffusion, therefore, given the involvement of matrix proteins in the regulation of expression of endothelial TJs, the basal lamina proteins are likely to maintain the diffusion as well (Tilling et al., 1998). The ECM and TJs are fundamental to the permeability of the BBB and the degradation of these proteins under pathological circumstances can potently exacerbate BBB dysfunction (Rascher et al., 2002). Additionally, matrix metalloproteinases (MMPs), particularly MMP-9, are capable of degrading the ECM and TJs, which enhances the permeability of the BBB and leads to vasogenic edema (Fujimoto et al., 2008; Halliday et al., 2013).

## NVU Dysfunction in the Pathophysiology of TBI

While numerous causes of trauma exist, TBI is consistently related to both primary and secondary damage mechanisms (Logsdon et al., 2015). During TBI, mechanical force directly leads to instant damage, which includes neuronal damage and vascular disruption, followed by the secondary injury mediated by succeeding pathophysiological processes such as oxidative stress, neuroinflammation, BBB dysfunction, and apoptosis (Abdul-Muneer et al., 2015; Chen X. et al., 2018; Khaksari et al., 2018; Main et al., 2018; Sevenich, 2018). All these primary and secondary pathologic processes contribute to the structural and/or metabolic abnormalities of the NVU, resulting in long-term neurological deficits in the patients (Zhang et al., 2005; Perez et al., 2017).

### Oxidative Stress

Oxidative stress gives rise to progressive neuropathology during TBI and contributes primarily to secondary injury (Abdul-Muneer et al., 2015; Anthonymuthu et al., 2018). Oxidative stress is considered physiological and biochemical stress or insult deriving mainly from reactive oxygen species (ROS) and reactive nitrogen species (RNS) (Abdul-Muneer et al., 2015). The enhanced production of ROS and RNS following TBI is due to excessive excitotoxicity and insufficient endogenous antioxidant systems. These reactive species result in increased oxidative stress and parallel the production of super-oxidized cellular and vascular structures, oxidized protein, cleaved DNA, and impaired mitochondrial electron transport chain, which can induce more ROS and RNS, thus triggering a vicious circle (Cornelius et al., 2013).

Oxidative stress and elevated toxic proteins can act on astrocytes to induce astrocyte-secreted pro-inflammatory factors, such as interleukin (IL)-6, monocyte chemoattractant protein (MCP)-1, and MMP-9, leading to the BBB compromise and neuroinflammation (Salminen et al., 2011). Oxidative stress is a major contributor to endothelial impairment. Enriched with mitochondria for the normal performance of ATP-dependent active transporters, endothelial cells can thus malfunction under increased oxidative stress and impaired mitochondrial functions (Cai et al., 2017b). Furthermore, oxidative stress and energy depletion induce the dysfunction of cellular ion channels that leads to the depolarization of neurons and the aggregation of excitatory neurotransmitters (e.g., glutamate), which further aggravate neuronal depolarization and the increase of toxic calcium levels (Weilinger et al., 2013). Moreover, enhanced oxidative stress and the subsequent biochemical cascade are adequate to induce early or late apoptosis, immediate cell necrosis and delayed neurodegeneration post TBI (Abdul-Muneer et al., 2015).

### Neuroinflammation

Neuroinflammation is a hallmark of different CNS pathologies (Sevenich, 2018). Following the initial injury, the changed microenvironment and released intracellular components from damaged cells trigger the activation and recruitment of local glial cells (Corps et al., 2015; Gyoneva and Ransohoff, 2015; Sharma et al., 2015). Both microglia and astrocytes react within 24 h and peak around day 3–7 post TBI (Fujita et al., 1998; Di Giovanni et al., 2005; Susarla et al., 2014). The activation and proliferation of glia release signaling factors and induce a robust, sterile immune reaction that consists of brain-resident and peripherally recruited inflammatory cells (Corps et al., 2015; Gyoneva and Ransohoff, 2015; Sharma et al., 2015). In addition to danger signals released by damaged cells, mitochondrial stress, glutamate excitotoxicity, and vascular injury have also been identified as inflammatory triggers of neuroinflammation (Simon et al., 2017). This immune reaction is supposed to exert a neuroprotective role and promote wound healing, but it can become neurodestructive and aggravate neuronal damage under certain circumstances (Murakami et al., 2011; Gyoneva and Ransohoff, 2015; Kumar et al., 2017). For instance, resident glia and infiltrating immune cells up-regulate the expression of tumor necrosis factor (TNF)-α, IL-6, and IL-1β rapidly to cope with injury, among which, however, TNF-α is correlated with BBB disruption and brain edema, and can act together with IL-1β to drive astrocyte dysfunction and resultant glutamate excitotoxicity (Ziebell and Morganti-Kossmann, 2010; Viviani et al., 2014; Simon et al., 2017). Notably, many mediators activated after TBI may exhibit pleiotropic effects in a context-dependent manner. In this regard, the conceptual framework of NVU may also help understand the shifting profiles of TBI pathophysiology over time (Lok et al., 2015).

As the inflammatory response progresses, the glial progenitor cells around the damaged tissue form a glial scar, which isolates the injured areas and inhibits the spread of inflammatory cells (Sofroniew, 2009; Karimi-Abdolrezaee and Billakanti, 2012; Burda and Sofroniew, 2014; Peng et al., 2014; Sofroniew, 2015). Nonetheless, the glial scar highly expresses inhibitory components for axonal regeneration and acts as both a physical and chemical barrier to axon elongation. The glial scar is therefore considered the main obstacle for axonal regeneration and the restoration of neuronal connectivity, which makes it a likely cause of the long-term sequelae in TBI patients (Voskuhl et al., 2009; Jeong et al., 2012; Cregg et al., 2014; Sharma et al., 2015).

### Blood–Brain Barrier Dysfunction

The BBB functions as a key homeostatic site between the CNS and other major systems in the body (Zhao et al., 2015). As the core feature of TBI, BBB dysfunction may indicate the severity of the injury as well as the length of recovery from TBI (Neuwelt et al., 2008; Main et al., 2018). The disruption of BBB integrity can result from the initial injury or arise secondarily from the succeeding pathological processes, including extensive inflammation, metabolic disturbances, and astrocytic dysfunction (Shlosberg et al., 2010; Badaut and Bix, 2014; Corps et al., 2015; Johnson et al., 2018). Following the brain injury, several pro-inflammatory pathways such as the IL-1β/nuclear factor-κ-gene binding (NF-κB) signaling pathway (Petty and Lo, 2002; Suzuki et al., 2010) and the Janus kinase/signal transducers and activators of transcription 1 (JAK/STAT1) signaling pathway (Gong et al., 2018) have been demonstrated to induce the upregulation of MMPs and the consequential degradation of the ECM and TJs. Moreover, upregulated NF-κB, as a transcription factor, also induces the transcription of pro-inflammatory genes and enzymes, including intracellular adhesion molecule-1 (ICAM-1) and inducible nitric oxide synthase (iNOS; Kumar et al., 2004). The former has been shown to increase BBB permeability through facilitating transendothelial leukocyte migration (Najjar et al., 2017) while the latter has been identified to be excitotoxic and neurotoxic as well as activate MMPs via nitric oxide (Ji et al., 2017; Chen H. et al., 2018). In addition, neuroinflammation and energy depletion result in the impairments of ionic transport, transporters, and mitochondrial oxidative metabolism of endothelial cells, which all exacerbate the breakdown of the BBB (Ott et al., 2007). Moreover, the BBB compromise can, in turn, aggravate the inflammatory response due to the enhanced influx of serum elements, proinflammatory molecules, and infiltrating leukocytes. It can also lead to cerebral hemorrhage, brain edema, and hypoxia (Shlosberg et al., 2010; Badaut and Bix, 2014; Corps et al., 2015; Johnson et al., 2018).

As described above, TJ-interconnected endothelial cells and pericytes are the main constituents that primarily contribute to the maintenance of the BBB (Armulik et al., 2010; Bell et al., 2010; Daneman et al., 2010; Cai et al., 2017b). The impairment of crosstalk between them ultimately leads to BBB dysfunction after TBI onset (Bhowmick et al., 2019). Astrocytes also hold a critical role in BBB function and cerebral water homeostasis (Burda et al., 2016) as astrocytic end-foot processes ensheathe the BBB and densely express perivascular aquaporin-4 (AQP4) channels (Louveau et al., 2017; Sweeney et al., 2018). In addition, BBB permeability can be altered by astrocyte-derived factors (Michinaga and Koyama, 2019). Moreover, other cell types involved in the NVU have also been shown to affect BBB homeostasis after TBI. For example, studies have indicated that oligodendrocyte precursor cells protect BBB against disruption via the transforming growth factor-β (TGF-β) signaling pathway (Seo et al., 2014). The activation, proliferation and phenotypic transformation of microglia indirectly have an impact on BBB function considering their involvement in the inflammatory response, especially the pro-inflammatory role (such as iNOS-expressing) of M1-like phenotype (Ladwig et al., 2017). These glial cells and microvascular cells have a functional interaction with each other in a paracrine manner (Abdul-Muneer et al., 2015). Therefore, the coordinated response of all components within the NVU is the determinant of BBB integrity (Lok et al., 2015).

### Persistent NVU Abnormalities and Imaging/Serum/CSF Biomarkers

Growing evidence has demonstrated these post-TBI NVU abnormalities may persist for months or years, especially in patients with a history of moderate-to-severe TBI (Hayes et al., 2017; LoBue et al., 2019). Reports regarding long-term brain changes after milder injuries have been mixed, and the results were often complicated by factors concerned with the number and severity/complications of injuries, genetic risk, and mental states and behavior (Hayes et al., 2017; LoBue et al., 2019). Diffuse axonal injury has been found in TBI of any severity and post-mortem studies reported widespread axonal pathology could continue for a sufficient length of time (weeks to months, even years) after mild-to-severe TBI in humans (Blumbergs et al., 1989; Johnson et al., 2013a, b; Shetty et al., 2014; Logsdon et al., 2015). A continuum of microglia activation across a wide range of TBI conditions was also observed in animal models (Nagamoto-Combs et al., 2007; Nagamoto-Combs et al., 2010) and human autopsy (form different cause) (Gentleman et al., 2004; Johnson et al., 2013a), even up to 18 years after TBI, and this was related to the consistently ongoing white matter degeneration (Johnson et al., 2013a). However, the functional impact of these microglia is still an enigma, as controversy remains regarding whether they exhibit a pro-inflammatory or pro-regenerative phenotype (Ramlackhansingh et al., 2011; Johnson et al., 2013a; Russo and McGavern, 2016; Scott et al., 2018; Tagge et al., 2018). Evidence of prolonged reactive astrogliosis after even mild injuries was revealed by brain tissue from animal models (Smith et al., 1997) and humans (Goldstein et al., 2012; Shively et al., 2017; Tagge et al., 2018) as well. Likewise, microvascular injury could be detected in the autopsy of long-term survivors from different types of TBI (Hay et al., 2015; Tagge et al., 2018). These continued NVU dysfunctions could bring about chronic pathological processes such as oxidative stress, chronic neuroinflammation, and proteinopathy, which were also reported to be identified years post TBI and could, in turn, aggravate the NVU dysfunction (Johnson et al., 2012; Simon et al., 2017; LoBue et al., 2019). Although chronic oxidative stress has not yet been observed following a single mild human TBI, it appears that oxidative stress after concussion and its associated pathophysiological processes may worsen or prolong symptoms (Hoffer et al., 2013; Cruz-Haces et al., 2017).

Currently, technological advances in clinical TBI neuroimaging make it possible to study NVU dysfunction and ensuing pathological processes *in vivo*, and findings by neuroimaging studies support the persistent abnormalities as well (LoBue et al., 2019). For example, microglia activation and related chronic neuroinflammation in moderate to severe TBI survivors can be assessed up to 17 years after trauma by positron emission tomography (PET) imaging technology binding to translocator protein, which is expressed by mitochondria of activated microglia (Folkersma et al., 2011; Ramlackhansingh et al., 2011). Moreover, significant increased binding to translocator protein can be identified by PET technology decades after the history of repetitive mild TBI in retired athletes compared to healthy controls (Coughlin et al., 2015). Similarly, selective PET techniques with ligands for neurodegenerative proteins [e.g., amyloid-β peptide (Aβ) and tau protein] can image the deposition of proteinopathy, showing increased binding in moderate-severe TBI victims/retired athletes versus control cases (Hong et al., 2014; Barrio et al., 2015; Scott et al., 2016). Advanced magnetic resonance imaging (MRI) modalities, such as diffusion tensor imaging (DTI, particularly in white matter pathology visualization), magnetic resonance spectroscopy (MRS, particularly in metabolic change assessment), susceptibility weight imaging (SWI, particularly in microscopic bleeding detection), and functional magnetic resonance imaging (fMRI, particularly in neurocognitive function examination) have emerged to allow the discovery of injury biomarkers and the detection of brain changes in patients with even mild TBI over time (Baugh et al., 2012; Xiong et al., 2014; Pavlovic et al., 2019). Additionally, Single Photon Emission Computed Tomography (SPECT) scan also has significance, as being a promising tool for detecting regional functional changes in the brain of patients sustaining TBI (Baugh et al., 2012).

Continued presence of some serum/plasma and CSF biomarkers is also able to persist many years post TBI, which depends on the type and extent of injury, thereby holding great promise for assessing the duration and degree of pathological processes (Williams et al., 2018; LoBue et al., 2019). Proteins including Aβ, tau, α-synuclein, and nuclear transactive response DNA-binding protein 43 (TDP-43) are critically implicated in the pathogenesis of several different neurodegenerative diseases, and have been utilized as neurobiological markers to monitor neuronal damage following TBI, moreover, different marker binding fingerprints may function as predictors for a particular late-onset neurodegenerative disease (Williams et al., 2018). A growing body of literature data has suggested that neurofilament light in the blood and/or CSF also serves as a biomarker indicating neuronal injury after TBI (Shahim et al., 2017; Bernick et al., 2018). Besides, glial fibrillary acidic protein, ubiquitin C-terminal hydrolase L1, S100β, and neuron-specific enolase have shown immense value as reliable blood/CSF markers in predicting poor outcome and evaluating microstructural injuries undetected by computed tomography (Bohmer et al., 2011; Mondello et al., 2016; Papa et al., 2016; Welch et al., 2016). Additionally, chronically elevated expression of serum cytokines may reflect chronic immune activation after TBI (Simon et al., 2017).

## Patients With TBI Have Increased Susceptibility to Neurodegenerative Diseases

Accumulating evidence provides support for an association between the risk of developing neurodegenerative disease and a prior TBI event. Previous studies have reported an increased risk of AD among TBI victims (Roberts et al., 1991; Fleminger et al., 2003). Similarly, 1.5–3.8 times higher incidence of PD has been reported in post-TBI cases (Goldman et al., 2006; Jafari et al., 2013). Traumatic brain injury, particularly of repeated TBI, is also considered a predisposing factor for ALS (Chen et al., 2007; Pupillo et al., 2018; Franz et al., 2019). Additionally, age at trauma has been suggested to be a determinant of the TBI victims’ susceptibility to ALS (Seals et al., 2016; Pupillo et al., 2018). Notably, although vulnerability to neurodegenerative diseases has been indicated in patients with TBI of any severity, this increased susceptibility is more common in survivors sustaining moderate-to-severe TBI (Hayes et al., 2017; Graham and Sharp, 2019; LoBue et al., 2019), and these patients have a 1.8-fold increase in neurodegenerative disease risk compared with those with mild TBI (Raj et al., 2017).

Unfortunately, mechanisms underlying this risk remain elusive thus far (LoBue et al., 2019). Early after the initial injury, TBI can induce the production of pathological proteins, whose neurotoxicity contributes directly to persistent abnormalities of brain structure and function, with these proteinopathies lasting for months or years (Goldstein et al., 2012; Johnson et al., 2012, 2013a; Hay et al., 2015; Williams et al., 2018). Intriguingly, post-TBI proteinopathies have similarities to multiple neurodegenerative diseases (Smith et al., 2013; Graham and Sharp, 2019). For instance, pathological accumulation of Aβ and neurofibrillary tangles comprising hyperphosphorylated neuronal tau brings about the development of AD (Zenaro et al., 2017), similarly, α-synuclein is linked to PD (Kalia and Lang, 2016). Furthermore, TDP-43 is considered the primary disease-related protein in ALS, and TDP-43 proteinopathy also features in other neurodegenerative disorders such as AD, PD, and Huntington’s disease (HD; Johnson et al., 2011). However, TDP-43 proteinopathy after TBI is more obscure and several controversial areas remain (Graham and Sharp, 2019). The study by Wiesner et al. (2018) addressed that brain trauma could boost ALS-related TDP-43 pathology, whereas the extent was modulated by ALS-related gene mutations, and the process was indicated to reversible and incapable of triggering ALS progression and neuronal vulnerability. Moreover, a single TBI does not appear to induce TDP-43 proteinopathy in humans (Johnson et al., 2011). In addition to proteinopathies, chronic neuroinflammation and persistent oxidative stress may represent other candidates for the increased susceptibility. Neuroinflammation is a hallmark of CNS disorders, including TBI and neurodegenerative diseases (Heneka et al., 2015; Russo and McGavern, 2016; Sevenich, 2018). Oxidative stress gives rise to progressive neuropathology and contributes primarily to the secondary injury post TBI (Abdul-Muneer et al., 2015; Anthonymuthu et al., 2018), concurrently being involved in the pathogenesis of neurodegenerative diseases (Cruz-Haces et al., 2017).

Taken collectively, proteinopathies, persistent oxidative stress, and chronic neuroinflammation may be the pivotal intermediary pathological processes between early and delayed post-TBI changes (LoBue et al., 2019). Herein we present that there is a pathological correlation between TBI and post-TBI neurodegenerative diseases, and we speculate it is the aforementioned persistent NVU dysfunction following TBI that accounts for the pathophysiological substrate and trigger condition for this pathological correlation. Readers seeking an in-depth discussion regarding the supporting evidence and internal mechanisms for this hypothesis should consult the section “Supporting Evidence and Precise Mechanisms.” But before that, as we have elucidated the bond between NVU dysfunction and TBI pathophysiology, however, is NVU dysfunction involved in the pathophysiology of neurodegenerative diseases?

## Is NVU Dysfunction Involved in the Pathophysiology of Neurodegenerative Diseases?

More and more lines of evidence favor the perspective that not only the neuronal degeneration and loss, but also aberrant neuroglia and vascular cells contribute to the pathogenesis of neurodegenerative diseases (Cai et al., 2017b). In this section, we describe several roles of NVU components involved in the pathophysiology of AD, PD, and ALS.

### Alzheimer’s Disease

A growing number of studies from post-mortem brain tissue reveal the existence of neurovascular dysfunction in patients with AD, including brain capillary leakage, degeneration of BBB-related cells (e.g., endothelial cells and pericytes), brain infiltration of peripheral cells, abnormal angiogenesis and molecular changes (Sweeney et al., 2018).

Blood–Brain Barrier disruption is considered the key pathway of AD onset (Cai et al., 2017b). This is firstly reflected in its impact on the aggregation of pathological protein. The dysfunction of NVU leads to aberrant cerebral blood flow regulation and impaired BBB transport, resulting in faulty Aβ clearance and increased Aβ production in soluble and fibrillary forms, which are the most neurotoxic and vasculotoxic forms (Benarroch, 2007). Accumulation of Aβ can subsequently induce oxidative stress, neuroinflammation and cell apoptosis, aggravating BBB impairment (Benarroch, 2007). Tau protein is associated with BBB compromise as well, as BBB dysfunction is in concert with the phenomenon that the major hippocampal blood vessels are surrounded by tau protein (Blair et al., 2015). In addition to pathological protein aggregation, AD is also pathologically characterized by chronic inflammation with the involvement of resident microglia and infiltrating peripheral immune cells (Heneka et al., 2015). A dysfunctional BBB is convenient for migrating immune cells to infiltrate the CNS. In this context, when the requisite adhesion molecules are expressed, circulating leukocytes migrate into the brain parenchyma through activated brain endothelial cells and then interact with NVU components to further affect their structural integrity and function (Zenaro et al., 2017).

### Parkinson’s Disease

In PD pathogenesis, BBB dysfunction is not as important as it is in AD, but the effects of neuronal and glial cell damage are more crucial. Dopaminergic neuronal loss and Lewy bodies and Lowy neurites containing α-synuclein are the hallmarks of PD (Kalia and Lang, 2016). Dopaminergic neurons are characterized as highly metabolic and thus have abundant amounts of mitochondria, the DNA of which is easily damaged by ROS and DNA repair can meanwhile be reduced in the absence of energy support (Liang et al., 2007; Gredilla et al., 2010). In addition, the metabolic process of dopamine itself will produce a large amount of accumulated ROS under pathological conditions (Cai et al., 2017b). Therefore, oxidative stress and energy depletion can easily give rise to dopaminergic neuronal loss as well as α-synuclein aggregation, which further forms Lewy bodies and Lowy neurites.

Besides neuronal loss, glial components in the NVU also play a pivotal role in PD onset. Equipped with unmyelinated axons, dopaminergic neurons interact most closely with astrocytes (Rodriguez et al., 2014; Rodriguez et al., 2015). In normal physiological conditions, astrocytes provide both structural and functional support for dopaminergic neurons in multifactorial ways (see section “Glial Components in the NVU” for the detailed mechanisms) while dysfunctional astrocytes secrete various cytokines, chemokines, and excitotoxins, which contribute to a series of pathological processes (Salminen et al., 2011; Zhou et al., 2020). Furthermore, microglia–astrocyte interactions can further propel disease progression (Chen et al., 2019).

### Amyotrophic Lateral Sclerosis

Amyotrophic lateral sclerosis is resulting from a complex combination of genetic predispositions and environmental insults. ALS-linked genes include *FUS*, *TARDBP* (the one encoding TDP-43), *SOD1*, *C9orf72*, among others (Sleigh et al., 2020). Based on the function of these genes, impaired cytoskeletal dynamics and axonal transport have emerged as a key role in ALS (Sleigh et al., 2020). Besides, dysfunctions of other NVU components are also involved in the pathogenesis of ALS. Both astrocytes and microglia have been argued to serve as central players in the pathogenesis. In the early stage of disease preceding the emergence of neuronal death and clinical symptoms, incompetent astrocytes due to astrodegeneration and astrocytic atrophy break the glutamate homeostasis and elicit glutamate excitotoxicity. Microglia are also induced to secrete neurotoxic factors, while in the later stage of disease, reactive astrogliosis and activated microglia may further promote neuronal damage and death (Verkhratsky et al., 2014). Additionally, the involvement of microvascular components in ALS pathology has also been suggested and microvascular compromise appears to precede the neuronal lesions and even the neuroinflammation (Guo and Lo, 2009).

## The Neurovascular Unit Dysfunction Hypothesis and its Limitations

### Definition and Relational Reasoning

The NVUD Hypothesis underscores the core role of NVU dysfunction in the development of post-TBI neurodegenerative diseases, which is concluded with relational reasoning from the following: (1) The NVU comprises neurons, neuroglial cells, vascular cells, and the basal lamina matrix of brain vasculature, playing critical roles in the homeostasis of the CNS, (2) all of the primary and secondary pathologic processes during TBI contribute to the persisting NVU dysfunction, including neuronal death, neuroglial dysfunction, and BBB compromise, (3) patients with TBI have an increased risk for developing neurodegenerative diseases, based on epidemiology and mutual pathological processes, and (4) NVU dysfunction is also involved in the pathophysiology of neurodegenerative diseases.

Additionally, we have mentioned in the previous sections that the persistent NVU abnormalities after TBI are related to the number and severity of injuries, and there is a dose–response relationship between TBI and later neurodegenerative disease. This similarity may further strengthen the reasonability of our assumption that continued NVU dysfunction serves as the pathophysiological substrate to trigger the development of post-TBI neurodegeneration.

**FIGURE 2:**
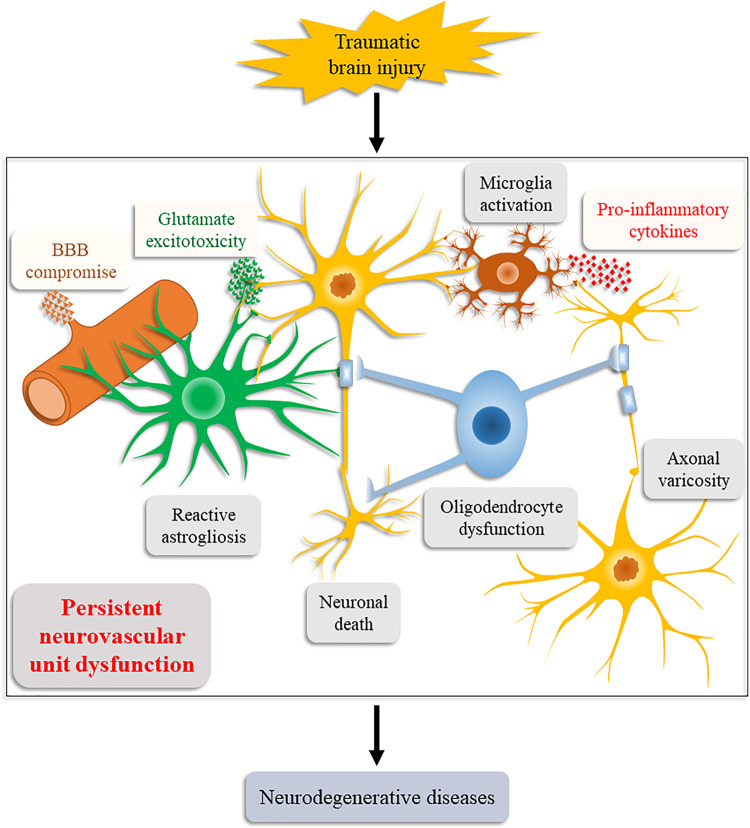
Schematic illustration of the Neurovascular Unit Dysfunction (NVUD) Hypothesis. During TBI, primary and secondary pathologic processes lead to persistent structural and/or functional abnormalities of the neurovascular unit, such as neuronal death, neuroglial dysfunction, and BBB compromise, which propel the progression of neurodegenerative diseases post TBI.

Consistent with our relational inference, increasing supporting evidence obtained by animal models and human postmortem studies has suggested NVU dysfunction an important causative factor for post-TBI neurodegeneration (Figure 2; Graham and Sharp, 2019; LoBue et al., 2019). These evidences also explain the induction of chronic neuroinflammation, persistent oxidative stress, and neurodegenerative protein aggregation after the initial injury.

### Supporting Evidence and Precise Mechanisms

Prolonged injured axons are considered a source of pathological proteins (Johnson et al., 2013b). Following TBI, the shear forces applied to the cytoskeleton result in damaged microstructure and impaired axonal transport (Maxwell et al., 2003; Tagge et al., 2018), which is also involved in AD (Johnson et al., 2013b) and ALS (Sleigh et al., 2020) pathogenesis. Impaired axonal transport can lead to the co-accumulation of amyloid precursor protein (APP) and AAP-cleaving enzymes in axonal varicosities within just hours of trauma and thus, forming abundant Aβ in the varicosities (Chen et al., 2004; Uryu et al., 2007; Chen et al., 2009; Tran et al., 2011). In the case where damaged axons eventually break down, these intraneuronal Aβ can aggregate in the parenchyma to form plaques (Chen et al., 2004; Johnson et al., 2013b). Shear forces also induce the dissociation of tau from microtubules, which is subsequently processed into a highly pathogenic tau form contributing to mitochondrial damage, neuronal apoptosis, and abnormal long-term potentiation (Kondo et al., 2015; Tagge et al., 2018). Besides, injured axons yield an extensive release of glutamate as well, coupled with cytokine stimulation, distorted glutamate receptor trafficking, and impaired glutamate clearance due to loss of astrocytic functional glutamate transporters, which together contribute to the dysregulation of glutamate homeostasis (Baker et al., 1993; Piao et al., 2019; Zhang et al., 2019). This dysregulation is adequate to incur neuroexcitotoxicity as well as resultant oxidative stress and mitochondrial dysfunction post TBI (Cornelius et al., 2013; Abdul-Muneer et al., 2015), which are strictly implicated in the PD pathogenesis including dopaminergic neuronal loss and α-synuclein aggregation (Liang et al., 2007; Gredilla et al., 2010; Cai et al., 2017b). Indeed, oxidative stress potentially plays a key role in protein carbonylation and pathological protein accumulation (Cruz-Haces et al., 2017). Notably, TBI is suggested to form a transmissible self-propagating proteinopathy (Zanier et al., 2018; Graham and Sharp, 2019). Altogether, the possibility of persistent axonopathy and transmissible proteinopathy provide a potential mechanism for long-term and extensive neurodegeneration in the brain, thereby propelling progression of neurodegenerative diseases (Johnson et al., 2012, 2013b; Zanier et al., 2018; Graham and Sharp, 2019).

Continued BBB dysfunction and neuroglial dysfunction following TBI are also implicated as triggers of post-TBI neurodegeneration, and the underlying mechanisms include, certainly, astrocyte dysfunction-induced impaired glutamate clearance as mentioned above. Perennial BBB alterations recruit brain-resident neuroglia and infiltrating leukocytes to form chronic neuroinflammation (Shetty et al., 2014; Hay et al., 2015), which is also a pathological characteristic of neurodegenerative diseases (Heneka et al., 2015; Sevenich, 2018). Similar to oxidative stress, neuroinflammation is indicated to elicit the abnormal accumulation of pathological proteins (LoBue et al., 2019), which may further exacerbate vascular lesion as the perivascular location of proteinopathies has been observed in autopsies of TBI victims (McKee et al., 2012; Tagge et al., 2018). The glymphatic system is a recently discovered brain-wide waste clearance pathway that utilizes astrocytic AQP4 channels to promote efficient elimination of interstitial solutes, including pathological proteins, from the CNS (Iliff et al., 2012; Jessen et al., 2015). In the acute phase following TBI, reactive astrogliosis is associated with alterations in AQP4 channel distribution and polarization, which are in line with the injury severity and persist for 14–28 days and 28 days in mild TBI and moderate TBI, respectively, resulting in decreased glymphatic influx and increased metabolic waste accumulation (Ren et al., 2013; Rasmussen et al., 2018; Sullan et al., 2018). Besides, decreased glymphatic function is also critically attributed to reactive astrogliosis-induced glial scars (Jessen et al., 2015). Furthermore, the glymphatic system functions primarily during sleep and is largely suppressed during waking state, whereas sleep deprivation constitutes one of the most frequently reported chronic complications following TBI (Jessen et al., 2015; Rasmussen et al., 2018; Sullan et al., 2018). As a result, the function of glymphatic pathway can be down to about 40% and maintained for at least 1 month after TBI (Iliff et al., 2014; Jessen et al., 2015). Therefore, both chronic impairment of BBB transport (Benarroch, 2007; Hay et al., 2015) and glymphatic pathway function (Iliff et al., 2014; Sullan et al., 2018) can induce impaired protein clearance, contributing to the succedent accumulation of neurotoxic and vasculotoxic proteins as well as the onset of post-TBI neurodegeneration.

### Neurovascular Unit Dysfunction Hypothesis as a Promising Theoretical Basis for Treatment

Neurovascular Unit Dysfunction abnormalities and ensuing pathological processes evolving over time have cast a spotlight on positive TBI interventions, which may be beneficial over an extended time frame (Ramlackhansingh et al., 2011). In the past, neuronal damage had been considered the main cause of functional impairment in brain injury or neurodegenerative diseases, and almost all the therapeutic strategies were targeted at repairing neuronal injury and rescuing neurons (Cai et al., 2017b). However, in most cases, saving neurons alone seemed to be insufficient for treating brain injuries or diseases (Lok et al., 2015). We have previously reviewed the advance of a variety of stem cells in TBI treatment. Although some preclinical studies and small trials showed a good therapeutic effect, practical value of clinic application has not reached a general success (Zhou et al., 2019). Analogously, stem cell therapies in other CNS diseases, including neurodegenerative diseases and stroke, have not generated substantial clinical improvements (Wang et al., 2017). The NVUD Hypothesis may provide new insights into the treatment strategies for TBI and potential prevention for later neurodegeneration, namely integrated restoration of all neural, neuroglial and vascular connectivity. Indeed, it is increasingly recognized that cell-cell crosstalk within the NVU components is absolutely required for remodeling and repair in acute brain injury and neurodegeneration (Lok et al., 2015). To date, several candidate agents have been found to promote the restoration of NVU integrity in TBI models, such as sonic hedgehog, and troxerutin cerebroprotein hydrolysate (Zhao et al., 2018; Wu et al., 2020), more preclinical and clinical studies are warranted.

### Limitations of NVUD Hypothesis

There are some aspects that NVUD Hypothesis cannot explain. For instance, studies have revealed that cell-cell signaling within the NVU is crucial in the pathogenesis of HD and that TDP-43 pathology may feature in HD (Gu et al., 2005; Shin et al., 2005; Gu et al., 2007; Faideau et al., 2010; Johnson et al., 2011; Jansen et al., 2017). However, the increased incidence of HD in TBI victims has been rarely reported. Moreover, despite universal occurred NVU dysfunction, only a subgroup of TBI victims develop late-onset neurodegenerative diseases, indicating other factors should be taken into consideration, such as genetic susceptibility (e.g., *APOE* genotyping) (Baugh et al., 2012; Goldstein et al., 2012; Shively et al., 2017; LoBue et al., 2019). Furthermore, aging not only increases the NVU’s vulnerability to neurodegeneration, but also attenuates its self-repair capabilities (Cai et al., 2017b; Wang et al., 2017). Thus, the crosstalk between NVUD Hypothesis and aging merits further explanation. The NVUD Hypothesis also needs further refinement. Although the NVU paradigm in this study focuses on the components surrounding the cerebral capillaries, critical roles of vascular components and perivascular nerve fibers connecting with larger blood vessels in CNS diseases have also been proposed (Zhang et al., 2012). These groups of structures may need to be incorporated into an expanded NVUD paradigm, which will revise the NVUD Hypothesis.

## Conclusion

In the present study, we propose the NVUD Hypothesis and discuss the reasonability. We also present its value as a promising theoretical basis for treatment and illustrate the limitations of this theory. The NVUD Hypothesis emphasizes that persistent NVU dysfunction functions as the pathophysiological substrate and trigger for late-onset neurodegeneration after TBI. Specifically, continued NVU abnormalities following TBI incur the chronic neuroinflammation, persistent oxidative stress, and neurodegenerative proteins aggregation, which in turn exacerbate NVU dysfunction and thus, forming a vicious circle and consequently leading to the progression of neurodegenerative diseases.

## Author Contributions

YZ and AS conceptualized the research project. YZ, YW, HW, SG, and YP wrote the manuscript and made the original figures. QC, AS, WX, JZ, and XD critically revised the texts and figures. AS supervised the research and led the discussion. All authors read and approved the final manuscript.

## Conflict of Interest

The authors declare that the research was conducted in the absence of any commercial or financial relationships that could be construed as a potential conflict of interest.

## Abbreviations

A β: amyloid β peptide
AD: Alzheimer’s disease
ALS: amyotrophic lateral sclerosis
AQP4: aquaporin-4
BBB: blood–brain barrier
CNS: central nervous system
DTI: diffusion tensor imaging
ECM: extracellular matrix
fMRI: functional magnetic resonance imaging
HD: Huntington disease
HTT: Huntingtin gene
ICAM-1: intracellular adhesion molecule-1
IL: interleukin
iNOS: inducible nitric oxide synthase
JAK: Janus kinase
MCP: monocyte chemoattractant protein
MMP: matrix metalloprotein
MRS: magnetic resonance spectroscopy
MSNs: medium spiny neurons
NF κ B: nuclear factor- κ B
NVU: neurovascular unit
NVUD: Neurovascular Unit Dysfunction
PD: Parkinson’s disease
PET: positron emission tomography
RNS: reactive nitrogen species
ROS: reactive oxygen species
SPECT: Single Photon Emission Computed Tomography
STAT1: signal transducers and activators of transcription 1 SWI susceptibility weight imaging
TBI: traumatic brain injury
TGF-β: transforming growth factor- β,TJ, tight junction
TNF: tumor necrosis factor.

## References

[B1] Abbott N. J. Ronnback L. Hansson E. (2006). Astrocyte-endothelial interactions at the blood-brain barrier. *Nat. Rev. Neurosci.* 7 41–53. 10.1038/nrn1824 16371949

[B2] Abdul-Muneer P. M. Chandra N. Haorah J. (2015). Interactions of oxidative stress and neurovascular inflammation in the pathogenesis of traumatic brain injury. *Mol. Neurobiol.* 51 966–979. 10.1007/s12035-014-8752-3 24865512 PMC9420084

[B3] Anderson C. M. Swanson R. A. (2000). Astrocyte glutamate transport: review of properties, regulation, and physiological functions. *Glia* 32 1–14. 10.1002/1098-1136(200010)32:1<1::aid-glia10>3.0.co;2-w 10975906

[B4] Anthonymuthu T. S. Kenny E. M. Lamade A. M. Kagan V. E. Bayir H. (2018). Oxidized phospholipid signaling in traumatic brain injury. *Free Rad. Biol. Med.* 124 493–503. 10.1016/j.freeradbiomed.2018.06.031 29964171 PMC6098726

[B5] Armulik A. Genove G. Mae M. Nisancioglu M. H. Wallgard E. Niaudet C. (2010). Pericytes regulate the blood-brain barrier. *Nature* 468 557–561. 20944627 10.1038/nature09522

[B6] Ascherio A. Schwarzschild M. A. (2016). The epidemiology of Parkinson’s disease: risk factors and prevention. *Lancet Neurol.* 15 1257–1272. 10.1016/s1474-4422(16)30230-7 27751556

[B7] Badaut J. Bix G. J. (2014). Vascular neural network phenotypic transformation after traumatic injury: potential role in long-term sequelae. *Transl. Stroke Res.* 5 394–406. 10.1007/s12975-013-0304-z 24323723 PMC4028405

[B8] Baker A. J. Moulton R. J. MacMillan V. H. Shedden P. M. (1993). Excitatory amino acids in cerebrospinal fluid following traumatic brain injury in humans. *J. Neurosurg.* 79 369–372. 10.3171/jns.1993.79.3.0369 8103092

[B9] Barrio J. R. Small G. W. Wong K. P. Huang S. C. Liu J. Merrill D. A. (2015). In vivo characterization of chronic traumatic encephalopathy using [F-18]FDDNP PET brain imaging. *Proc. Natl. Acad. Sci. U.S.A.* 112 E2039–E2047. 25848027 10.1073/pnas.1409952112 PMC4413350

[B10] Barshikar S. Bell K. R. (2017). Sleep disturbance after TBI. *Curr. Neurol. Neurosci. Rep.* 17:87. 10.1007/s11910-017-0792-4 28933033

[B11] Baugh C. M. Stamm J. M. Riley D. O. Gavett B. E. Shenton M. E. Lin A. (2012). Chronic traumatic encephalopathy: neurodegeneration following repetitive concussive and subconcussive brain trauma. *Brain Imag. Behav.* 6 244–254. 10.1007/s11682-012-9164-5 22552850

[B12] Bell R. D. Winkler E. A. Sagare A. P. Singh I. LaRue B. Deane R. (2010). Pericytes control key neurovascular functions and neuronal phenotype in the adult brain and during brain aging. *Neuron* 68 409–427. 10.1016/j.neuron.2010.09.043 21040844 PMC3056408

[B13] Benarroch E. E. (2007). Neurovascular unit dysfunction: a vascular component of Alzheimer disease? *Neurology* 68 1730–1732. 10.1212/01.wnl.0000264502.92649.ab 17502556

[B14] Bernick C. Zetterberg H. Shan G. Banks S. Blennow K. (2018). Longitudinal performance of plasma neurofilament light and tau in professional fighters: the professional fighters brain health study. *J. Neurotr.* 35 2351–2356. 10.1089/neu.2017.5553 29609512

[B15] Bhowmick S. D’Mello V. Caruso D. Wallerstein A. Abdul-Muneer P. M. (2019). Impairment of pericyte-endothelium crosstalk leads to blood-brain barrier dysfunction following traumatic brain injury. *Exp. Neurol.* 317 260–270. 10.1016/j.expneurol.2019.03.014 30926390

[B16] Blair L. J. Frauen H. D. Zhang B. Nordhues B. A. Bijan S. Lin Y. C. (2015). Tau depletion prevents progressive blood-brain barrier damage in a mouse model of tauopathy. *Acta Neuropathol. Commun.* 3:8. 10.1186/s40478-015-0186-2 PMC4353464 25775028

[B17] Blumbergs P. C. Jones N. R. North J. B. (1989). Diffuse axonal injury in head trauma. *J. Neurol. Neurosurg. Psychiatry* 52 838–841. 2769276 10.1136/jnnp.52.7.838 PMC1031929

[B18] Boespflug E. L. Iliff J. J. (2018). The emerging relationship between interstitial fluid-cerebrospinal fluid exchange, amyloid-beta, and sleep. *Biol. Psychiatry* 83 328–336. 10.1016/j.biopsych.2017.11.031 29279202 PMC5767516

[B19] Bohmer A. E. Oses J. P. Schmidt A. P. Peron C. S. Krebs C. L. Oppitz P. P. (2011). Neuron-specific enolase, S100B, and glial fibrillary acidic protein levels as outcome predictors in patients with severe traumatic brain injury. *Neurosurgery* 68 1624–1630. 21368691 10.1227/NEU.0b013e318214a81f

[B20] Burda J. E. Bernstein A. M. Sofroniew M. V. (2016). Astrocyte roles in traumatic brain injury. *Exp. Neurol.* 275(Pt 3), 305–315. 10.1016/j.expneurol.2015.03.020 25828533 PMC4586307

[B21] Burda J. E. Sofroniew M. V. (2014). Reactive gliosis and the multicellular response to CNS damage and Disease. *Neuron* 81 229–248. 10.1016/j.neuron.2013.12.034 24462092 PMC3984950

[B22] Cai W. Liu H. Zhao J. Chen L. Y. Chen J. Lu Z. (2017a). Pericytes in brain injury and repair after ischemic stroke. *Transl. Stroke Res.* 8 107–121. 10.1007/s12975-016-0504-4 27837475 PMC5350040

[B23] Cai W. Zhang K. Li P. Zhu L. Xu J. Yang B. (2017b). Dysfunction of the neurovascular unit in ischemic stroke and neurodegenerative diseases: an aging effect. *Ageing Res. Rev.* 34 77–87. 10.1016/j.arr.2016.09.006 27697546 PMC5384332

[B24] Chen H. Guan B. Chen X. Chen X. Li C. Qiu J. (2018). Baicalin attenuates blood-brain barrier disruption and hemorrhagic transformation and improves neurological outcome in ischemic stroke rats with delayed t-PA treatment: involvement of ONOO(-)-MMP-9 pathway. *Transl. Stroke Res.* 9 515–529. 10.1007/s12975-017-0598-3 29275501

[B25] Chen X. Pan Z. Fang Z. Lin W. Wu S. Yang F. (2018). Omega-3 polyunsaturated fatty acid attenuates traumatic brain injury-induced neuronal apoptosis by inducing autophagy through the upregulation of SIRT1-mediated deacetylation of Beclin-1. *J. Neuroinflamm.* 15:310. 10.1186/s12974-018-1345-8 PMC6225685 30409173

[B26] Castriotta R. J. Wilde M. C. Lai J. M. Atanasov S. Masel B. E. Kuna S. T. (2007). Prevalence and consequences of sleep disorders in traumatic brain injury. *J. Clin. Sleep Med.* 3 349–356. 10.5664/jcsm.26855 17694722 PMC1978308

[B27] Chen H. Richard M. Sandler D. P. Umbach D. M. Kamel F. (2007). Head injury and amyotrophic lateral sclerosis. *Am. J. Epidemiol.* 166 810–816. 17641152 10.1093/aje/kwm153 PMC2239342

[B28] Chen X. H. Johnson V. E. Uryu K. Trojanowski J. Q. Smith D. H. (2009). A lack of amyloid beta plaques despite persistent accumulation of amyloid beta in axons of long-term survivors of traumatic brain injury. *Brain Pathol.* 19 214–223. 10.1111/j.1750-3639.2008.00176.x 18492093 PMC3014260

[B29] Chen X. H. Siman R. Iwata A. Meaney D. F. Trojanowski J. Q. Smith D. H. (2004). Long-term accumulation of amyloid-beta, beta-secretase, presenilin-1, and caspase-3 in damaged axons following brain trauma. *Am. J. Pathol.* 165 357–371. 10.1016/s0002-9440(10)63303-2 15277212 PMC1618579

[B30] Chen Z. Zhong D. Li G. (2019). The role of microglia in viral encephalitis: a review. *J. Neuroinflamm.* 16:76. 10.1186/s12974-019-1443-2 PMC6454758 30967139

[B31] Colangelo A. M. Cirillo G. Lavitrano M. L. Alberghina L. Papa M. (2012). Targeting reactive astrogliosis by novel biotechnological strategies. *Biotechnol. Adv.* 30 261–271. 10.1016/j.biotechadv.2011.06.016 21763415

[B32] Cordone S. Annarumma L. Rossini P. M. De Gennaro L. (2019). Sleep and beta-Amyloid deposition in Alzheimer Disease: insights on mechanisms and possible innovative treatments. *Front. Pharmacol.* 10:695. 10.3389/fphar.2019.00695 31281257 PMC6595048

[B33] Cornelius C. Crupi R. Calabrese V. Graziano A. Milone P. Pennisi G. (2013). Traumatic brain injury: oxidative stress and neuroprotection. *Antioxid. Redox Signal.* 19 836–853. 23547621 10.1089/ars.2012.4981

[B34] Corps K. N. Roth T. L. McGavern D. B. (2015). Inflammation and neuroprotection in traumatic brain injury. *JAMA Neurol.* 72 355–362. 25599342 10.1001/jamaneurol.2014.3558 PMC5001842

[B35] Coughlin J. M. Wang Y. Munro C. A. Ma S. Yue C. Chen S. (2015). Neuroinflammation and brain atrophy in former NFL players: an in vivo multimodal imaging pilot study. *Neurobiol. Dis.* 74 58–65. 10.1016/j.nbd.2014.10.019 25447235 PMC4411636

[B36] Cregg J. M. DePaul M. A. Filous A. R. Lang B. T. Tran A. Silver J. (2014). Functional regeneration beyond the glial scar. *Exp. Neurol.* 253 197–207. 10.1016/j.expneurol.2013.12.024 24424280 PMC3951813

[B37] Cruz-Haces M. Tang J. Acosta G. Fernandez J. Shi R. (2017). Pathological correlations between traumatic brain injury and chronic neurodegenerative diseases. *Transl. Neurodegen.* 6:20. 10.1186/s40035-017-0088-2 PMC5504572 28702179

[B38] Danbolt N. C. (2001). Glutamate uptake. *Prog. Neurobiol.* 65 1–15. 11369436 10.1016/s0301-0082(00)00067-8

[B39] Daneman R. Zhou L. Kebede A. A. Barres B. A. (2010). Pericytes are required for blood-brain barrier integrity during embryogenesis. *Nature* 468 562–566. 10.1038/nature09513 20944625 PMC3241506

[B40] de la Tremblaye P. B. O’Neil D. A. LaPorte M. J. Cheng J. P. Beitchman J. A. Thomas T. C. (2018). Elucidating opportunities and pitfalls in the treatment of experimental traumatic brain injury to optimize and facilitate clinical translation. *Neurosci. Biobehav. Rev.* 85 160–175. 10.1016/j.neubiorev.2017.05.022 28576511 PMC5709241

[B41] de Lau L. M. L. Breteler M. M. B. (2006). Epidemiology of Parkinson’s disease. *Lancet Neurol.* 5 525–535. 16713924 10.1016/S1474-4422(06)70471-9

[B42] Di Giovanni S. Movsesyan V. Ahmed F. Cernak I. Schinelli S. Stoica B. (2005). Cell cycle inhibition provides neuroprotection and reduces glial proliferation and scar formation after traumatic brain injury. *Proc. Natl. Acad. Sci. U.S.A.* 102 8333–8338. 10.1073/pnas.0500989102 15923260 PMC1149422

[B43] Dringen R. Gutterer J. M. Hirrlinger J. (2000). Glutathione metabolism in brain metabolic interaction between astrocytes and neurons in the defense against reactive oxygen species. *Eur. J. Biochem.* 267 4912–4916. 10.1046/j.1432-1327.2000.01597.x 10931173

[B44] Faideau M. Kim J. Cormier K. Gilmore R. Welch M. Auregan G. (2010). In vivo expression of polyglutamine-expanded huntingtin by mouse striatal astrocytes impairs glutamate transport: a correlation with Huntington’s disease subjects. *Hum. Mol. Genet.* 19 3053–3067. 10.1093/hmg/ddq212 20494921 PMC2901144

[B45] Fleminger S. Oliver D. L. Lovestone S. Rabe-Hesketh S. Giora A. (2003). Head injury as a risk factor for Alzheimer’s disease: the evidence 10 years on, a partial replication. *J. Neurol. Neurosurg. Psychiatry* 74 857–862. 10.1136/jnnp.74.7.857 12810767 PMC1738550

[B46] Folkersma H. Boellaard R. Yaqub M. Kloet R. W. Windhorst A. D. Lammertsma A. A. (2011). Widespread and prolonged increase in (R)-(11)C-PK11195 binding after traumatic brain injury. *J. Nuclear Med.* 52 1235–1239. 10.2967/jnumed.110.084061 21764792

[B47] Fotuhi M. Hachinski V. Whitehouse P. J. (2009). Changing perspectives regarding late-life dementia. *Nat. Rev. Neurol.* 5 649–658. 10.1038/nrneurol.2009.175 19918254

[B48] Franz C. K. Joshi D. Daley E. L. Grant R. A. Dalamagkas K. Leung A. (2019). Impact of traumatic brain injury on amyotrophic lateral sclerosis: from bedside to bench. *J. Neurophysiol.* 122 1174–1185. 10.1152/jn.00572.2018 31116639 PMC6766734

[B49] Fujimoto M. Takagi Y. Aoki T. Hayase M. Marumo T. Gomi M. (2008). Tissue inhibitor of metalloproteinases protect blood-brain barrier disruption in focal cerebral ischemia. *J. Cereb. Blood Flow Metab.* 28 1674–1685. 10.1038/jcbfm.2008.59 18560439

[B50] Fujita T. Yoshimine T. Maruno M. Hayakawa T. (1998). Cellular dynamics of macrophages and microglial cells in reaction to stab wounds in rat cerebral cortex. *Acta Neurochir.* 140 275–279. 10.1007/s007010050095 9638265

[B51] GBD 2016 Traumatic Brain Injury and Spinal Cord Injury Collaborators (2019). Global, regional, and national burden of traumatic brain injury and spinal cord injury, 1990-2016: a systematic analysis for the Global burden of disease study 2016. *Lancet Neurol.* 18 56–87. 30497965 10.1016/S1474-4422(18)30415-0 PMC6291456

[B52] Gentleman S. M. Leclercq P. D. Moyes L. Graham D. I. Smith C. Griffin W. S. (2004). Long-term intracerebral inflammatory response after traumatic brain injury. *Forensic Sci. Int.* 146 97–104. 10.1016/j.forsciint.2004.06.027 15542269

[B53] Goldman S. M. Tanner C. M. Oakes D. Bhudhikanok G. S. Gupta A. Langston J. W. (2006). Head injury and Parkinson’s disease risk in twins. *Ann. Neurol.* 60 65–72. 10.1002/ana.20882 16718702

[B54] Goldstein L. E. Fisher A. M. Tagge C. A. Zhang X. L. Velisek L. Sullivan J. A. (2012). Chronic traumatic encephalopathy in blast-exposed military veterans and a blast neurotrauma mouse model. *Sci. Transl. Med.* 4:134ra60. 10.1126/scitranslmed.3003716 PMC3739428 22593173

[B55] Gong L. Manaenko A. Fan R. Huang L. Enkhjargal B. McBride D. (2018). Osteopontin attenuates inflammation via JAK2/STAT1 pathway in hyperglycemic rats after intracerebral hemorrhage. *Neuropharmacology* 138 160–169. 10.1016/j.neuropharm.2018.06.009 29885817 PMC6487497

[B56] Graham N. S. Sharp D. J. (2019). Understanding neurodegeneration after traumatic brain injury: from mechanisms to clinical trials in dementia. *J. Neurol. Neurosurg. Psychiatry* 90 1221–1233. 10.1136/jnnp-2017-317557 31542723 PMC6860906

[B57] Gredilla R. Bohr V. A. Stevnsner T. (2010). Mitochondrial DNA repair and association with aging–an update. *Exp. Gerontol.* 45 478–488. 10.1016/j.exger.2010.01.017 20096766 PMC2879476

[B58] Gu X. Andre V. M. Cepeda C. Li S. H. Li X. J. Levine M. S. (2007). Pathological cell-cell interactions are necessary for striatal pathogenesis in a conditional mouse model of Huntington’s disease. *Mol. Neurodegen.* 2:8. 10.1186/1750-1326-2-8 17470275 PMC1885431

[B59] Gu X. Li C. Wei W. Lo V. Gong S. Li S. H. (2005). Pathological cell-cell interactions elicited by a neuropathogenic form of mutant Huntingtin contribute to cortical pathogenesis in HD mice. *Neuron* 46 433–444. 10.1016/j.neuron.2005.03.025 15882643

[B60] Guo S. Lo E. H. (2009). Dysfunctional cell-cell signaling in the neurovascular unit as a paradigm for central nervous system disease. *Stroke* 40 S4–S7. 19064781 10.1161/STROKEAHA.108.534388 PMC3712844

[B61] Gyoneva S. Ransohoff R. M. (2015). Inflammatory reaction after traumatic brain injury: therapeutic potential of targeting cell-cell communication by chemokines. *Trends Pharmacol. Sci.* 36 471–480. 10.1016/j.tips.2015.04.003 25979813 PMC4485943

[B62] Hailer N. P. (2008). Immunosuppression after traumatic or ischemic CNS damage: it is neuroprotective and illuminates the role of microglial cells. *Prog. Neurobiol.* 84 211–233. 10.1016/j.pneurobio.2007.12.001 18262323

[B63] Halliday M. R. Pomara N. Sagare A. P. Mack W. J. Frangione B. Zlokovic B. V. (2013). Relationship between cyclophilin a levels and matrix metalloproteinase 9 activity in cerebrospinal fluid of cognitively normal apolipoprotein e4 carriers and blood-brain barrier breakdown. *JAMA Neurol.* 70 1198–1200. 24030206 10.1001/jamaneurol.2013.3841 PMC4047029

[B64] Hay J. R. Johnson V. E. Young A. M. Smith D. H. Stewart W. (2015). Blood-brain barrier disruption is an early event that may persist for many years after traumatic brain injury in humans. *J. Neuropathol. Exp. Neurol.* 74 1147–1157. 10.1093/jnen/74.12.1147 26574669 PMC8744142

[B65] Hayes J. P. Logue M. W. Sadeh N. Spielberg J. M. Verfaellie M. Hayes S. M. (2017). Mild traumatic brain injury is associated with reduced cortical thickness in those at risk for Alzheimer’s disease. *Brain* 140 813–825. 28077398 10.1093/brain/aww344 PMC6075586

[B66] Heneka M. T. Carson M. J. El Khoury J. Landreth G. E. Brosseron F. Feinstein D. L. (2015). Neuroinflammation in Alzheimer’s disease. *Lancet Neurol.* 14 388–405. 25792098 10.1016/S1474-4422(15)70016-5 PMC5909703

[B67] Hoffer M. E. Balaban C. Slade M. D. Tsao J. W. Hoffer B. (2013). Amelioration of acute sequelae of blast induced mild traumatic brain injury by N-acetyl cysteine: a double-blind, placebo controlled study. *PLoS One* 8:e54163 10.1371/journal.pone.054163 PMC3553161 23372680

[B68] Hong Y. T. Veenith T. Dewar D. Outtrim J. G. Mani V. Williams C. (2014). Amyloid imaging with carbon 11-labeled Pittsburgh compound B for traumatic brain injury. *JAMA Neurol.* 71 23–31. 24217171 10.1001/jamaneurol.2013.4847 PMC4084932

[B69] Hu X. Liou A. K. Leak R. K. Xu M. An C. Suenaga J. (2014). Neurobiology of microglial action in CNS injuries: receptor-mediated signaling mechanisms and functional roles. *Prog. Neurobiol.* 119-120 60–84. 10.1016/j.pneurobio.2014.06.002 24923657 PMC4121732

[B70] Hynes R. O. (1992). Integrins: versatility, modulation, and signaling in cell adhesion. *Cell* 69 11–25. 10.1016/0092-8674(92)90115-s 1555235

[B71] Iliff J. J. Chen M. J. Plog B. A. Zeppenfeld D. M. Soltero M. Yang L. (2014). Impairment of glymphatic pathway function promotes tau pathology after traumatic brain injury. *J. Neurosci.* 34 16180–16193. 10.1523/jneurosci.3020-14.2014 25471560 PMC4252540

[B72] Iliff J. J. Wang M. Liao Y. Plogg B. A. Peng W. Gundersen G. A. (2012). A paravascular pathway facilitates CSF flow through the brain parenchyma and the clearance of interstitial solutes, including amyloid beta. *Sci. Transl. Med.* 4:147ra111. 10.1126/scitranslmed.3003748 22896675 PMC3551275

[B73] Jafari S. Etminan M. Aminzadeh F. Samii A. (2013). Head injury and risk of Parkinson disease: a systematic review and meta-analysis. *Mov. Disord.* 28 1222–1229. 10.1002/mds.25458 23609436

[B74] Jansen A. H. van Hal M. Op den Kelder I. C. Meier R. T. de Ruiter A. A. Schut M. H. (2017). Frequency of nuclear mutant huntingtin inclusion formation in neurons and glia is cell-type-specific. *Glia* 65 50–61. 10.1002/glia.23050 27615381 PMC5129569

[B75] Jayakumar A. R. Norenberg M. D. (2010). The Na–K–Cl Co-transporter in astrocyte swelling. *Metab. Brain Dis.* 25 31–38. 10.1007/s11011-010-9180-3 20336356

[B76] Jeong S. R. Kwon M. J. Lee H. G. Joe E. H. Lee J. H. Kim S. S. (2012). Hepatocyte growth factor reduces astrocytic scar formation and promotes axonal growth beyond glial scars after spinal cord injury. *Exp. Neurol.* 233 312–322. 10.1016/j.expneurol.2011.10.021 22079829

[B77] Jessen N. A. Munk A. S. Lundgaard I. Nedergaard M. (2015). The glymphatic system: a beginner’s guide. *Neurochem. Res.* 40 2583–2599. 25947369 10.1007/s11064-015-1581-6 PMC4636982

[B78] Ji B. Zhou F. Han L. Yang J. Fan H. Li S. (2017). Sodium tanshinone IIA sulfonate enhances effectiveness Rt-PA treatment in acute ischemic stroke patients associated with ameliorating blood-brain barrier damage. *Transl. Stroke Res.* 8 334–340. 10.1007/s12975-017-0526-6 28243834 PMC5493726

[B79] Johnson V. E. Stewart J. E. Begbie F. D. Trojanowski J. Q. Smith D. H. Stewart W. (2013a). Inflammation and white matter degeneration persist for years after a single traumatic brain injury. *Brain* 136 28–42. 10.1093/brain/aws322 23365092 PMC3562078

[B80] Johnson V. E. Stewart W. Smith D. H. (2013b). Axonal pathology in traumatic brain injury. *Exp. Neurol.* 246 35–43. 10.1016/j.expneurol.2012.01.013 22285252 PMC3979341

[B81] Johnson V. E. Stewart W. Smith D. H. (2012). Widespread tau and amyloid-beta pathology many years after a single traumatic brain injury in humans. *Brain Pathol.* 22 142–149. 10.1111/j.1750-3639.2011.00513.x 21714827 PMC3979351

[B82] Johnson V. E. Stewart W. Trojanowski J. Q. Smith D. H. (2011). Acute and chronically increased immunoreactivity to phosphorylation-independent but not pathological TDP-43 after a single traumatic brain injury in humans. *Acta Neuropathol.* 122 715–726. 10.1007/s00401-011-0909-9 22101322 PMC3979333

[B83] Johnson V. E. Weber M. T. Xiao R. Cullen D. K. Meaney D. F. Stewart W. (2018). Mechanical disruption of the blood-brain barrier following experimental concussion. *Acta Neuropathol.* 135 711–726. 10.1007/s00401-018-1824-0 29460006 PMC6532777

[B84] Kalia L. V. Lang A. E. (2016). Parkinson disease in 2015: evolving basic, pathological and clinical concepts in PD. *Nat. Rev. Neurol.* 12 65–66. 10.1038/nrneurol.2015.249 26782330

[B85] Kardos J. Heja L. Jemnitz K. Kovacs R. Palkovits M. (2017). The nature of early astroglial protection-Fast activation and signaling. *Prog. Neurobiol.* 153 86–99. 10.1016/j.pneurobio.2017.03.005 28342942

[B86] Karimi-Abdolrezaee S. Billakanti R. (2012). Reactive astrogliosis after spinal cord injury-beneficial and detrimental effects. *Mol. Neurobiol.* 46 251–264. 10.1007/s12035-012-8287-4 22684804

[B87] Khaksari M. Soltani Z. Shahrokhi N. (2018). Effects of female sex steroids administration on pathophysiologic mechanisms in traumatic brain injury. *Transl. Stroke Res.* 9 393–416. 10.1007/s12975-017-0588-5 29151229

[B88] Kofuji P. Newman E. A. (2004). Potassium buffering in the central nervous system. *Neuroscience* 129 1045–1056. 15561419 10.1016/j.neuroscience.2004.06.008 PMC2322935

[B89] Kondo A. Shahpasand K. Mannix R. Qiu J. Moncaster J. Chen C. H. (2015). Antibody against early driver of neurodegeneration cis P-tau blocks brain injury and tauopathy. *Nature* 523 431–436. 10.1038/nature14658 26176913 PMC4718588

[B90] Kumar A. Nisha C. M. Silakari C. Sharma I. Anusha K. Gupta N. (2016). Current and novel therapeutic molecules and targets in Alzheimer’s disease. *J. Form. Med. Assoc.* 115 3–10. 10.1016/j.jfma.2015.04.001 26220908

[B91] Kumar A. Stoica B. A. Loane D. J. Yang M. Abulwerdi G. Khan N. (2017). Microglial-derived microparticles mediate neuroinflammation after traumatic brain injury. *J. Neuroinflamm.* 14:47. 10.1186/s12974-017-0819-4 PMC5351060 28292310

[B92] Kumar A. Takada Y. Boriek A. M. Aggarwal B. B. (2004). Nuclear factor-kappaB: its role in health and disease. *J. Mol. Med.* 82 434–448. 15175863 10.1007/s00109-004-0555-y

[B93] Ladwig A. Walter H. L. Hucklenbroich J. Willuweit A. Langen K. J. Fink G. R. (2017). Osteopontin augments M2 microglia response and separates M1- and M2-polarized microglial activation in permanent focal cerebral ischemia. *Med. Inflamm.* 2017:7189421. 10.1155/2017/7189421 PMC5632451 29104378

[B94] Lang F. Busch G. L. Ritter M. Völkl H. Waldegger S. Gulbins E. (1998). Functional significance of cell volume regulatory mechanisms. *Physiol. Rev.* 78 247–306. 10.1152/physrev.1998.78.1.247 9457175

[B95] Li C. Ebrahimi A. Schluesener H. (2013). Drug pipeline in neurodegeneration based on transgenic mice models of Alzheimer’s disease. *Ageing Res. Rev.* 12 116–140. 10.1016/j.arr.2012.09.002 22982398

[B96] Liang C. L. Wang T. T. Luby-Phelps K. German D. C. (2007). Mitochondria mass is low in mouse substantia nigra dopamine neurons: implications for Parkinson’s disease. *Exp. Neurol.* 203 370–380. 10.1016/j.expneurol.2006.08.015 17010972

[B97] Lo E. H. Rosenberg G. A. (2009). The neurovascular unit in health and disease: introduction. *Stroke* 40 S2–S3. 19064779 10.1161/STROKEAHA.108.534404 PMC2811575

[B98] LoBue C. Munro C. Schaffert J. Didehbani N. Hart J. Batjer H. (2019). Traumatic brain injury and risk of long-term brain changes, accumulation of pathological markers, and developing dementia: a review. *J. Alzheimer Dis.* 70 629–654. 10.3233/jad-190028 31282414

[B99] Logsdon A. F. Lucke-Wold B. P. Turner R. C. Huber J. D. Rosen C. L. Simpkins J. W. (2015). Role of microvascular disruption in brain damage from traumatic brain injury. *Comprehens. Physiol.* 5 1147–1160. 10.1002/cphy.c140057 26140712 PMC4573402

[B100] Lok J. Wang X. S. Xing C. H. Maki T. K. Wu L. M. Guo S. Z. (2015). Targeting the neurovascular unit in brain trauma. *CNS Neurosci. Therap.* 21 304–308. 10.1111/cns.12359 25475543 PMC6495706

[B101] Louveau A. Plog B. A. Antila S. Alitalo K. Nedergaard M. Kipnis J. (2017). Understanding the functions and relationships of the glymphatic system and meningeal lymphatics. *J. Clin. Invest.* 127 3210–3219. 10.1172/jci90603 28862640 PMC5669566

[B102] Luna J. Diagana M. Ait Aissa L. Tazir M. Ali Pacha L. Kacem I. (2019). Clinical features and prognosis of amyotrophic lateral sclerosis in Africa: the TROPALS study. *J. Neurol. Neurosurg. Psychiatry* 90 20–29. 30242088 10.1136/jnnp-2018-318469

[B103] Maas A. I. R. Menon D. K. Adelson P. D. Andelic N. Bell M. J. Belli A. (2017). Traumatic brain injury: integrated approaches to improve prevention, clinical care, and research. *Lancet Neurol.* 16 987–1048. 29122524 10.1016/S1474-4422(17)30371-X

[B104] Magistretti P. J. (2006). Neuron-glia metabolic coupling and plasticity. *J. Exp. Biol.* 209 2304–2311. 10.1242/jeb.02208 16731806

[B105] Magistretti P. J. Pellerin L. (1999). Cellular mechanisms of brain energy metabolism and their relevance to functional brain imaging. *Philos. Trans. R. Soc. Lond. B Biol. Sci.* 354 1155–1163. 10.1098/rstb.1999.0471 10466143 PMC1692634

[B106] Main B. S. Villapol S. Sloley S. S. Barton D. J. Parsadanian M. Agbaegbu C. (2018). Apolipoprotein E4 impairs spontaneous blood brain barrier repair following traumatic brain injury. *Mol. Neurodegen.* 13:17. 10.1186/s13024-018-0249-5 PMC5885297 29618365

[B107] Martland H. S. (1928). Punch drunk. *J. Am. Med. Assoc.* 91 1103–1107.

[B108] Masters C. L. Bateman R. Blennow K. Rowe C. C. Sperling R. A. Cummings J. L. (2015). Alzheimer’s disease. *Nat. Rev. Dis. Prim.* 1:15056. 10.1038/nrdp.2015.56 27188934

[B109] Maxwell W. L. Domleo A. McColl G. Jafari S. S. Graham D. I. (2003). Post-acute alterations in the axonal cytoskeleton after traumatic axonal injury. *J. Neurotrauma* 20 151–168. 10.1089/08977150360547071 12675969

[B110] McKee A. C. Stein T. D. Nowinski C. J. Stern R. A. Daneshvar D. H. Alvarez V. E. (2012). The spectrum of disease in chronic traumatic encephalopathy. *Brain* 136 43–64. 23208308 10.1093/brain/aws307 PMC3624697

[B111] Mendiola-Precoma J. Berumen L. C. Padilla K. Garcia-Alcocer G. (2016). Therapies for prevention and treatment of Alzheimer’s Disease. *Biomed. Res. Intern.* 2016:2589276. 10.1155/2016/2589276 PMC4980501 27547756

[B112] Michinaga S. Koyama Y. (2019). Dual roles of astrocyte-derived factors in regulation of blood-brain barrier function after brain damage. *Intern. J. Mol. Sci.* 20:E571. 10.3390/ijms20030571 PMC6387062 30699952

[B113] Mondello S. Kobeissy F. Vestri A. Hayes R. L. Kochanek P. M. Berger R. P. (2016). Serum concentrations of ubiquitin C-terminal hydrolase-L1 and glial fibrillary acidic protein after pediatric traumatic brain injury. *Sci. Rep.* 6:28203. 10.1038/srep28203 PMC4913316 27319802

[B114] Mortimer J. A. Van Duijn C. M. Chandra V. Fratiglioni L. Graves A. B. Heyman A. (1991). Head trauma as a risk factor for alzheimer’s disease: A collaborativere-analysis of case-control studies. *Int. J. Epidemiol.* 20 S28–S35. 1833351 10.1093/ije/20.supplement_2.s28

[B115] Murakami K. Koide M. Dumont T. M. Russell S. R. Tranmer B. I. Wellman G. C. (2011). Subarachnoid hemorrhage induces gliosis and increased expression of the pro-inflammatory cytokine high mobility group box 1 protein. *Transl. Stroke Res.* 2 72–79. 10.1007/s12975-010-0052-2 21479116 PMC3072171

[B116] Nagamoto-Combs K. McNeal D. W. Morecraft R. J. Combs C. K. (2007). Prolonged microgliosis in the rhesus monkey central nervous system after traumatic brain injury. *J. Neurotrauma* 24 1719–1742. 10.1089/neu.2007.0377 18001202

[B117] Nagamoto-Combs K. Morecraft R. J. Darling W. G. Combs C. K. (2010). Long-term gliosis and molecular changes in the cervical spinal cord of the rhesus monkey after traumatic brain injury. *J. Neurotrauma* 27 565–585. 10.1089/neu.2009.0966 20030560 PMC2867631

[B118] Najjar S. Pahlajani S. De Sanctis V. Stern J. N. H. Najjar A. Chong D. (2017). Neurovascular unit dysfunction and blood-brain barrier hyperpermeability contribute to schizophrenia neurobiology: a theoretical integration of clinical and experimental evidence. *Front. Psychiatry* 8:83. 10.3389/fpsyt.2017.00083 28588507 PMC5440518

[B119] Neuwelt E. Abbott N. J. Abrey L. Banks W. A. Blakley B. Davis T. (2008). Strategies to advance translational research into brain barriers. *Lancet Neurol.* 7 84–96. 10.1016/s1474-4422(07)70326-5 18093565

[B120] Orimo S. (2017). New development of diagnosis and treatment for Parkinson’s disease. *Clin. Neurol.* 57 259–273. 10.5692/clinicalneurol.cn-000969 28552865

[B121] Ott M. Gogvadze V. Orrenius S. Zhivotovsky B. (2007). Mitochondria, oxidative stress and cell death. *Apoptosis* 12 913–922. 10.1007/s10495-007-0756-2 17453160

[B122] Papa L. Mittal M. K. Ramirez J. Ramia M. Kirby S. Silvestri S. (2016). In children and youth with mild and moderate traumatic brain injury, glial fibrillary acidic protein out-performs s100beta in detecting traumatic intracranial lesions on computed tomography. *J. Neurotrauma* 33 58–64. 10.1089/neu.2015.3869 25752485 PMC4700391

[B123] Pavlovic D. Pekic S. Stojanovic M. Popovic V. (2019). Traumatic brain injury: neuropathological, neurocognitive and neurobehavioral sequelae. *Pituitary* 22 270–282. 10.1007/s11102-019-00957-9 30929221

[B124] Peng L. Parpura V. Verkhratsky A. (2014). Editorial neuroglia as a central element of neurological diseases: an underappreciated target for therapeutic intervention. *Curr. Neuropharmacol.* 12 303–307. 10.2174/1570159x12999140829152550 25342938 PMC4207070

[B125] Perez E. J. Tapanes S. A. Loris Z. B. Balu D. T. Sick T. J. Coyle J. T. (2017). Enhanced astrocytic d-serine underlies synaptic damage after traumatic brain injury. *J. Clin. Invest.* 127 3114–3125. 10.1172/jci92300 28714867 PMC5531405

[B126] Petty M. A. Lo E. H. (2002). Junctional complexes of the blood-brain barrier: permeability changes in neuroinflammation. *Prog. Neurobiol.* 68 311–323. 10.1016/s0301-0082(02)00128-4 12531232

[B127] Piao C. S. Holloway A. L. Hong-Routson S. Wainwright M. S. (2019). Depression following traumatic brain injury in mice is associated with down-regulation of hippocampal astrocyte glutamate transporters by thrombin. *J. Cereb. Blood Flow Metab.* 39 58–73. 10.1177/0271678x17742792 29135354 PMC6311670

[B128] Plemel J. R. Keough M. B. Duncan G. J. Sparling J. S. Yong V. W. Stys P. K. (2014). Remyelination after spinal cord injury: is it a target for repair? *Prog. Neurobiol.* 117 54–72. 24582777 10.1016/j.pneurobio.2014.02.006

[B129] Pupillo E. Poloni M. Bianchi E. Giussani G. Logroscino G. Zoccolella S. (2018). Trauma and amyotrophic lateral sclerosis: a european population-based case-control study from the EURALS consortium. *Amyotrop. Later. Scler. Frontotemp. Degen.* 19 118–125. 10.1080/21678421.2017.1386687 29063790

[B130] Raj R. Kaprio J. Korja M. Mikkonen E. D. Jousilahti P. Siironen J. (2017). Risk of hospitalization with neurodegenerative disease after moderate-to-severe traumatic brain injury in the working-age population: a retrospective cohort study using the Finnish national health registries. *PLoS Med.* 14:e1002316 10.1371/journal.pone.01002316 PMC5497945 28678790

[B131] Ramlackhansingh A. F. Brooks D. J. Greenwood R. J. Bose S. K. Turkheimer F. E. Kinnunen K. M. (2011). Inflammation after trauma: microglial activation and traumatic brain injury. *Ann. Neurol.* 70 374–383. 10.1002/ana.22455 21710619

[B132] Rascher G. Fischmann A. Kroger S. Duffner F. Grote E. H. Wolburg H. (2002). Extracellular matrix and the blood-brain barrier in glioblastoma multiforme: spatial segregation of tenascin and agrin. *Acta Neuropathol.* 104 85–91. 10.1007/s00401-002-0524-x 12070669

[B133] Rasmussen M. K. Mestre H. Nedergaard M. (2018). The glymphatic pathway in neurological disorders. *Lancet Neurol.* 17 1016–1024. 10.1016/s1474-4422(18)30318-1 30353860 PMC6261373

[B134] Ren Z. Iliff J. J. Yang L. Yang J. Chen X. Chen M. J. (2013). ‘Hit & Run’ model of closed-skull traumatic brain injury (TBI) reveals complex patterns of post-traumatic AQP4 dysregulation. *J. Cereb. Blood Flow Metab.* 33 834–845. 23443171 10.1038/jcbfm.2013.30 PMC3677112

[B135] Roberts G. W. Gentleman S. M. Lynch A. Graham D. I. (1991). beta A4 amyloid protein deposition in brain after head trauma. *Lancet* 338 1422–1423. 10.1016/0140-6736(91)92724-g 1683421

[B136] Rodriguez M. Morales I. Rodriguez-Sabate C. Sanchez A. Castro R. Brito J. M. (2014). The degeneration and replacement of dopamine cells in Parkinson’s disease: the role of aging. *Front. Neuroanat.* 8:80 10.3389/fphar.2019.0080 PMC4124707 25147507

[B137] Rodriguez M. Rodriguez-Sabate C. Morales I. Sanchez A. Sabate M. (2015). Parkinson’s disease as a result of aging. *Aging Cell* 14 293–308. 25677794 10.1111/acel.12312 PMC4406659

[B138] Rothstein J. D. Dykes-Hoberg M. Pardo C. A. Bristol L. A. Jin L. Kuncl R. W. (1996). Knockout of glutamate transporters reveals a major role for astroglial transport in excitotoxicity and clearance of glutamate. *Neuron* 16 675–686. 10.1016/s0896-6273(00)80086-0 8785064

[B139] Russo M. V. McGavern D. B. (2016). Inflammatory neuroprotection following traumatic brain injury. *Science* 353 783–785. 10.1126/science.aaf6260 27540166 PMC5260471

[B140] Salminen A. Ojala J. Kaarniranta K. Haapasalo A. Hiltunen M. Soininen H. (2011). Astrocytes in the aging brain express characteristics of senescence-associated secretory phenotype. *Eur. J. Neurosci.* 34 3–11. 10.1111/j.1460-9568.2011.07738.x 21649759

[B141] Scott G. Ramlackhansingh A. F. Edison P. Hellyer P. Cole J. Veronese M. (2016). Amyloid pathology and axonal injury after brain trauma. *Neurology* 86 821–828. 10.1212/wnl.0000000000002413 26843562 PMC4793784

[B142] Scott G. Zetterberg H. Jolly A. Cole J. H. De Simoni S. Jenkins P. O. (2018). Minocycline reduces chronic microglial activation after brain trauma but increases neurodegeneration. *Brain* 141 459–471. 10.1093/brain/awx339 29272357 PMC5837493

[B143] Seals R. M. Hansen J. Gredal O. Weisskopf M. G. (2016). Physical trauma and amyotrophic lateral sclerosis: a population-based study using danish national registries. *Am. J. Epidemiol.* 183 294–301. 10.1093/aje/kwv169 26825926 PMC4753279

[B144] Seo J. H. Maki T. Maeda M. Miyamoto N. Liang A. C. Hayakawa K. (2014). Oligodendrocyte precursor cells support blood-brain barrier integrity via TGF-beta signaling. *PLoS One* 9:e103174. 10.1371/journal.pone.0103174 25078775 PMC4117639

[B145] Sevenich L. (2018). Brain-resident microglia and blood-borne macrophages orchestrate central nervous system inflammation in neurodegenerative disorders and brain cancer. *Front. Immunol.* 9:697. 10.3389/fimmu.2018.00697 PMC5897444 29681904

[B146] Shahim P. Zetterberg H. Tegner Y. Blennow K. (2017). Serum neurofilament light as a biomarker for mild traumatic brain injury in contact sports. *Neurology* 88 1788–1794. 10.1212/wnl.0000000000003912 28404801 PMC5419986

[B147] Sharma K. Zhang G. Li S. (2015). *Chapter 11 - Astrogliosis and Axonal Regeneration.* Oxford: Academic Press.

[B148] Shetty A. K. Mishra V. Kodali M. Hattiangady B. (2014). Blood brain barrier dysfunction and delayed neurological deficits in mild traumatic brain injury induced by blast shock waves. *Front. Cell. Neurosci.* 8:232. 10.3389/fphar.2019.00232 25165433 PMC4131244

[B149] Shin J. Y. Fang Z. H. Yu Z. X. Wang C. E. Li S. H. Li X. J. (2005). Expression of mutant huntingtin in glial cells contributes to neuronal excitotoxicity. *J. Cell Biol.* 171 1001–1012. 10.1083/jcb.200508072 16365166 PMC2171327

[B150] Shively S. B. Edgerton S. L. Iacono D. Purohit D. P. Qu B. X. Haroutunian V. (2017). Localized cortical chronic traumatic encephalopathy pathology after single, severe axonal injury in human brain. *Acta Neuropathol.* 133 353–366. 10.1007/s00401-016-1649-7 27885490 PMC5325841

[B151] Shlosberg D. Benifla M. Kaufer D. Friedman A. (2010). Blood-brain barrier breakdown as a therapeutic target in traumatic brain injury. *Nat. Rev. Neurol.* 6 393–403. 10.1038/nrneurol.2010.74 20551947 PMC3625732

[B152] Shokri-Kojori E. Wang G. J. Wiers C. E. Demiral S. B. Guo M. Kim S. W. (2018). beta-Amyloid accumulation in the human brain after one night of sleep deprivation. *Proc. Natl. Acad. Sci. U.S.A.* 115 4483–4488. 10.1073/pnas.1721694115 29632177 PMC5924922

[B153] Simon D. W. McGeachy M. J. Bayir H. Clark R. S. Loane D. J. Kochanek P. M. (2017). The far-reaching scope of neuroinflammation after traumatic brain injury. *Nat. Rev. Neurol.* 13 171–191. 10.1038/nrneurol.2017.13 28186177 PMC5675525

[B154] Sleigh J. N. Tosolini A. P. Gordon D. Devoy A. Fratta P. Fisher E. M. C. (2020). Mice carrying ALS mutant TDP-43, but not mutant FUS, display in vivo defects in axonal transport of signaling endosomes. *Cell Rep.* 30 3655–3662. 32187538 10.1016/j.celrep.2020.02.078 PMC7090381

[B155] Slemmer J. E. Shacka J. J. Sweeney M. I. Weber J. T. (2008). Antioxidants and free radical scavengers for the treatment of stroke, traumatic brain injury and aging. *Curr. Med. Chem.* 15 404–414. 10.2174/092986708783497337 18288995

[B156] Smith D. H. Chen X. H. Pierce J. E. Wolf J. A. Trojanowski J. Q. Graham D. I. (1997). Progressive atrophy and neuron death for one year following brain trauma in the rat. *J. Neurotrauma* 14 715–727. 10.1089/neu.1997.14.715 9383090

[B157] Smith D. H. Johnson V. E. Stewart W. (2013). Chronic neuropathologies of single and repetitive TBI: substrates of dementia? *Nat. Rev. Neurol.* 9 211–221. 10.1038/nrneurol.2013.29 23458973 PMC4513655

[B158] Sofroniew M. V. (2009). dissection of reactive astrogliosis and glial scar formation. *Trends Neurosci.* 32 638–647. 10.1016/j.tins.2009.08.002 19782411 PMC2787735

[B159] Sofroniew M. V. (2015). Astrocyte barriers to neurotoxic inflammation. *Nat. Rev. Neurosci.* 16 249–263. 10.1038/nrn3898 25891508 PMC5253239

[B160] Sofroniew M. V. Vinters H. V. (2010). Astrocytes: biology and pathology. *Acta Neuropathol.* 119 7–35. 10.1007/s00401-009-0619-8 20012068 PMC2799634

[B161] Sullan M. J. Asken B. M. Jaffee M. S. DeKosky S. T. Bauer R. M. (2018). Glymphatic system disruption as a mediator of brain trauma and chronic traumatic encephalopathy. *Neurosci. Biobehav. Rev.* 84 316–324. 10.1016/j.neubiorev.2017.08.016 28859995

[B162] Susarla B. T. Villapol S. Yi J. H. Geller H. M. Symes A. J. (2014). Temporal patterns of cortical proliferation of glial cell populations after traumatic brain injury in mice. *ASN Neuro* 6 159–170. 24670035 10.1042/AN20130034 PMC4013687

[B163] Suzuki H. Ayer R. Sugawara T. Chen W. Sozen T. Hasegawa Y. (2010). Protective effects of recombinant osteopontin on early brain injury after subarachnoid hemorrhage in rats. *Crit. Care Med.* 38 612–618. 10.1097/ccm.0b013e3181c027ae 19851092 PMC2808465

[B164] Sweeney M. D. Ayyadurai S. Zlokovic B. V. (2016). Pericytes of the neurovascular unit: key functions and signaling pathways. *Nat. Neurosci.* 19 771–783. 10.1038/nn.4288 27227366 PMC5745011

[B165] Sweeney M. D. Sagare A. P. Zlokovic B. V. (2018). Blood-brain barrier breakdown in Alzheimer disease and other neurodegenerative disorders. *Nat. Rev. Neurol.* 14 133–150. 10.1038/nrneurol.2017.188 29377008 PMC5829048

[B166] Tagge C. A. Fisher A. M. Minaeva O. V. Gaudreau-Balderrama A. Moncaster J. A. Zhang X. L. (2018). Concussion, microvascular injury, and early tauopathy in young athletes after impact head injury and an impact concussion mouse model. *Brain* 141 422–458. 10.1093/brain/awx350 29360998 PMC5837414

[B167] Tilling T. Engelbertz C. Decker S. Korte D. Huwel S. Galla H. J. (2002). Expression and adhesive properties of basement membrane proteins in cerebral capillary endothelial cell cultures. *Cell Tissue Res.* 310 19–29. 10.1007/s00441-002-0604-1 12242480

[B168] Tilling T. Korte D. Hoheisel D. Galla H. J. (1998). Basement membrane proteins influence brain capillary endothelial barrier function in vitro. *J. Neurochem.* 71 1151–1157. 10.1046/j.1471-4159.1998.71031151.x 9721740

[B169] Tran H. T. LaFerla F. M. Holtzman D. M. Brody D. L. (2011). Controlled cortical impact traumatic brain injury in 3xTg-AD mice causes acute intra-axonal amyloid-beta accumulation and independently accelerates the development of tau abnormalities. *J. Neurosci.* 31 9513–9525. 10.1523/jneurosci.0858-11.2011 21715616 PMC3146343

[B170] Tso M. K. Macdonald R. L. (2014). Subarachnoid hemorrhage: a review of experimental studies on the microcirculation and the neurovascular unit. *Transl. Stroke Res.* 5 174–189. 10.1007/s12975-014-0323-4 24510780

[B171] Uryu K. Chen X. H. Martinez D. Browne K. D. Johnson V. E. Graham D. I. (2007). Multiple proteins implicated in neurodegenerative diseases accumulate in axons after brain trauma in humans. *Exp. Neurol.* 208 185–192. 10.1016/j.expneurol.2007.06.018 17826768 PMC3979356

[B172] Verkhratsky A. Parpura V. Pekna M. Pekny M. Sofroniew M. (2014). Glia in the pathogenesis of neurodegenerative diseases. *Biochem. Soc. Trans.* 42 1291–1301. 10.1042/bst20140107 25233406

[B173] Viviani B. Boraso M. Marchetti N. Marinovich M. (2014). Perspectives on neuroinflammation and excitotoxicity: a neurotoxic conspiracy? *Neurotoxicology* 43 10–20. 10.1016/j.neuro.2014.03.004 24662010

[B174] Voskuhl R. R. Peterson R. S. Song B. Ao Y. Morales L. B. Tiwari-Woodruff S. (2009). Reactive astrocytes form scar-like perivascular barriers to leukocytes during adaptive immune inflammation of the CNS. *J. Neurosci.* 29 11511–11522. 10.1523/jneurosci.1514-09.2009 19759299 PMC2768309

[B175] Walz W. (2000). Role of astrocytes in the clearance of excess extracellular potassium. *Neurochem. Int.* 36 291–300. 10.1016/s0197-0186(99)00137-0 10732996

[B176] Wang Y. Ji X. Leak R. K. Chen F. Cao G. (2017). Stem cell therapies in age-related neurodegenerative diseases and stroke. *Ageing Res. Rev.* 34 39–50. 10.1016/j.arr.2016.11.002 27876573 PMC5250574

[B177] Weilinger N. L. Maslieieva V. Bialecki J. Sridharan S. S. Tang P. L. Thompson R. J. (2013). Ionotropic receptors and ion channels in ischemic neuronal death and dysfunction. *Acta Pharmacol. Sin.* 34 39–48. 10.1038/aps.2012.95 22864302 PMC4086487

[B178] Welch R. D. Ayaz S. I. Lewis L. M. Unden J. Chen J. Y. Mika V. H. (2016). Ability of serum glial fibrillary acidic protein, Ubiquitin C-terminal hydrolase-L1, and S100B to differentiate normal and abnormal head computed tomography findings in patients with suspected mild or moderate traumatic brain injury. *J. Neurotrauma* 33 203–214. 10.1089/neu.2015.4149 26467555 PMC4722555

[B179] Wiesner D. Tar L. Linkus B. Chandrasekar A. Olde Heuvel F. Dupuis L. (2018). Reversible induction of TDP-43 granules in cortical neurons after traumatic injury. *Exp. Neurol.* 299 15–25. 10.1016/j.expneurol.2017.09.011 28941811

[B180] Williams S. M. Peltz C. Yaffe K. Schulz P. Sierks M. R. (2018). CNS disease-related protein variants as blood-based biomarkers in traumatic brain injury. *Neurology* 91 702–709. 10.1212/wnl.0000000000006322 30297502 PMC6177276

[B181] Wimo A. Guerchet M. Ali G.-C. Wu Y.-T. Prina A. M. Winblad B. (2017). The worldwide costs of dementia 2015 and comparisons with 2010. *Alzheimer Dement.* 13 1–7. 10.1016/j.jalz.2016.07.150 27583652 PMC5232417

[B182] Wu J. He J. Tian X. Zhong J. Li H. Sun X. (2020). Activation of the hedgehog pathway promotes recovery of neurological function after traumatic brain injury by protecting the neurovascular unit. *Transl. Stroke Res.* 10.1007/s12975-019-00771-2 [Epub ahead of print]. 31898187

[B183] Xiong K. L. Zhu Y. S. Zhang W. G. (2014). Diffusion tensor imaging and magnetic resonance spectroscopy in traumatic brain injury: a review of recent literature. *Brain Imag. Behav.* 8 487–496. 10.1007/s11682-013-9288-2 24449140

[B184] Yang Z. Wang K. K. (2015). Glial fibrillary acidic protein: from intermediate filament assembly and gliosis to neurobiomarker. *Trends Neurosci.* 38 364–374. 10.1016/j.tins.2015.04.003 25975510 PMC4559283

[B185] Ye Z. C. Wyeth M. S. Baltan-Tekkok S. Ransom B. R. (2003). hemichannels in astrocytes: a novel mechanism of glutamate release. *J. Neurosci.* 23 3588–3596. 10.1523/jneurosci.23-09-03588.2003 12736329 PMC6742182

[B186] Zanier E. R. Bertani I. Sammali E. Pischiutta F. Chiaravalloti M. A. Vegliante G. (2018). Induction of a transmissible tau pathology by traumatic brain injury. *Brain* 141 2685–2699. 30084913 10.1093/brain/awy193 PMC6113646

[B187] Zenaro E. Piacentino G. Constantin G. (2017). The blood-brain barrier in Alzheimer’s disease. *Neurobiol. Dis.* 107 41–56. 27425887 10.1016/j.nbd.2016.07.007 PMC5600438

[B188] Zhang D. Xiao M. Wang L. Jia W. (2019). Blood-based glutamate scavengers reverse traumatic brain injury-induced synaptic plasticity disruption by decreasing glutamate level in hippocampus interstitial fluid, but not cerebral spinal fluid, in vivo. *Neurotox. Res.* 35 360–372. 10.1007/s12640-018-9961-8 30255425

[B189] Zhang J. H. Badaut J. Tang J. Obenaus A. Hartman R. Pearce W. J. (2012). The vascular neural network–a new paradigm in stroke pathophysiology. *Nat. Rev. Neurol.* 8 711–716. 10.1038/nrneurol.2012.210 23070610 PMC3595043

[B190] Zhang X. Chen Y. Jenkins L. W. Kochanek P. M. Clark R. S. (2005). Bench-to-bedside review: apoptosis/programmed cell death triggered by traumatic brain injury. *Crit. Care* 9 66–75. 15693986 10.1186/cc2950 PMC1065095

[B191] Zhao H. Liu Y. Zeng J. Li D. Huang Y. (2018). *Troxerutin cerebroprotein* hydrolysate injection ameliorates neurovascular injury induced by traumatic brain injury - via endothelial nitric oxide synthase pathway regulation. *Int. J. Neurosci.* 128 1118–1127. 10.1080/00207454.2018.1486828 29883225

[B192] Zhao Z. Nelson A. R. Betsholtz C. Zlokovic B. V. (2015). Establishment and dysfunction of the blood-brain barrier. *Cell* 163 1064–1078. 10.1016/j.cell.2015.10.067 26590417 PMC4655822

[B193] Zhou Y. Shao A. Xu W. Wu H. Deng Y. (2019). Advance of stem cell treatment for traumatic brain injury. *Front. Cell. Neurosci.* 13:301. 10.3389/fphar.2019.00301 31456663 PMC6700304

[B194] Zhou Y. Shao A. Yao Y. Tu S. Deng Y. Zhang J. (2020). Dual roles of astrocytes in plasticity and reconstruction after traumatic brain injury. *Cell Commun. Signal.* 18:62. 10.1186/s12964-020-00549-2 PMC7158016 32293472

[B195] Ziebell J. M. Morganti-Kossmann M. C. (2010). Involvement of pro- and anti-inflammatory cytokines and chemokines in the pathophysiology of traumatic brain injury. *Neurotherapeutics* 7 22–30. 10.1016/j.nurt.2009.10.016 20129494 PMC5084109

[B196] Zou J. Wang Y. X. Dou F. F. Lu H. Z. Ma Z. W. Lu P. H. (2010). Glutamine synthetase down-regulation reduces astrocyte protection against glutamate excitotoxicity to neurons. *Neurochem. Int.* 56 577–584. 10.1016/j.neuint.2009.12.021 20064572 PMC2831119

